# Activation of Latent Courtship Circuitry in the Brain of *Drosophila* Females Induces Male-like Behaviors

**DOI:** 10.1016/j.cub.2016.07.021

**Published:** 2016-09-26

**Authors:** Carolina Rezával, Siddharth Pattnaik, Hania J. Pavlou, Tetsuya Nojima, Birgit Brüggemeier, Luis A.D. D’Souza, Hany K.M. Dweck, Stephen F. Goodwin

**Affiliations:** 1 Centre for Neural Circuits and Behaviour, University of Oxford, Tinsley Building, Mansfield Road, Oxford OX1 3SR, UK; 2 Department of Evolutionary Neuroethology, Max Planck Institute for Chemical Ecology, 07745 Jena, Germany

## Abstract

Courtship in *Drosophila melanogaster* offers a powerful experimental paradigm for the study of innate sexually dimorphic behaviors [1, 2]. Fruit fly males exhibit an elaborate courtship display toward a potential mate [1, 2]. Females never actively court males, but their response to the male’s display determines whether mating will actually occur. Sex-specific behaviors are hardwired into the nervous system via the actions of the sex determination genes *doublesex* (*dsx*) and *fruitless* (*fru*) [1]. Activation of male-specific *dsx*/*fru*^+^ P1 neurons in the brain initiates the male’s courtship display [3, 4], suggesting that neurons unique to males trigger this sex-specific behavior. In females, *dsx*^*+*^ neurons play a pivotal role in sexual receptivity and post-mating behaviors [1, 2, 5, 6, 7, 8, 9]. Yet it is still unclear how *dsx*^+^ neurons and dimorphisms in these circuits give rise to the different behaviors displayed by males and females. Here, we manipulated the function of *dsx*^+^ neurons in the female brain to investigate higher-order neurons that drive female behaviors. Surprisingly, we found that activation of female *dsx*^+^ neurons in the brain induces females to behave like males by promoting male-typical courtship behaviors. Activated females display courtship toward conspecific males or females, as well other *Drosophila* species. We uncovered specific *dsx*^+^ neurons critical for driving male courtship and identified pheromones that trigger such behaviors in activated females. While male courtship behavior was thought to arise from male-specific central neurons, our study shows that the female brain is equipped with latent courtship circuitry capable of inducing this male-specific behavioral program.

## Highlights

• Activation of brain *dsx*-pC1 neurons promote male-like courtship in females
• Activated females court conspecific males and females and other *Drosophila* species
• Methyl pheromones trigger male courtship behaviors in activated females
• The female brain is equipped with latent circuitry underlying male-like behavior

## Teaser

Rezával et al. found that activation of specific neurons in the brain induces female fruit flies to display male-like courtship behaviors and identified pheromones that induce such behaviors. Thus, this study shows that the female fly brain is equipped with latent courtship circuitry that is capable of inducing a male-specific behavioral program.

## Results and Discussion

### Brain-Restricted Activation of *dsx*^+^ Neurons Induces Male Courtship Behaviors in Females

**Figure 1:**
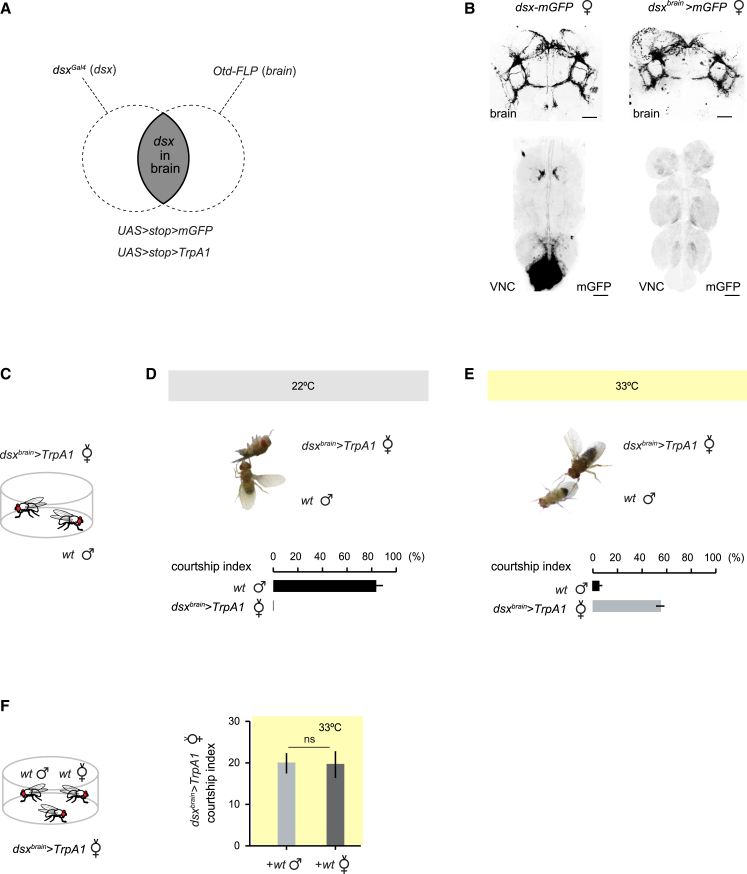
Activation of *dsx*^*+*^ Neurons in the Female Brain Elicits Male-Typical Courtship Behaviors (A) Intersectional strategy used to isolate *dsx*^*+*^ neurons in the female brain. Brain-specific flippase (*Otd-FLP)* is used in combination with the *dsx*-specific driver *dsx*^*Gal4*^ and either *UAS*>*stop*>*mCD8::GFP* (mGFP) or *UAS*>*stop*>*TrpA1* (TrpA). (B) Confocal images showing membrane-bound GFP expression in all *dsx*^*+*^ neurons of the brain and VNC of females (*dsx-mGFP*; left) or intersected *dsx*^*+*^ neurons in the brain (*dsx*^*brain*^>*mGFP*; right). GFP staining is shown in black. Scale bars, 50 μm. (C–E) Thermoactivation of *dsx*^*+*^ neurons in the female brain. (C) Courtship assay schematic: a *Otd-FLP/UAS*>*stop*>*TrpA1;dsx*^*Gal4*^/+ virgin female (*dsx*^*brain*^>*TrpA1*) was placed in a chamber with a wild-type (*wt*) male at the control (22°C) or activating temperature (33°C). (D and E) Courtship assays for *dsx*^*brain*^>*TrpA1* virgin females paired with wild-type males at 22°C (D) or 33°C (E). The courtship indices displayed by both males and females are reported. Courtship levels of wild-type males at 22°C are significantly higher than at 33°C and courtship levels of *dsx*^*brain*^>*TrpA1* females at 33°C are significantly higher than at 22°C (p < 0.0001; Mann-Whitney test). n = 20. (F) *dsx*^*brain*^>*TrpA1* females do not display a sexual preference. Schematic of the courtship preference assay: a *dsx*^*brain*^>*TrpA1* female was placed in a chamber with a male and a female at 33°C (left). Percentage of time a *dsx*^*brain*^>*TrpA1* virgin female spent courting the male or female at 33°C (right). n = 20. A Mann-Whitney test was performed. ns, not significant. Error bars, SEM. See also Figure S1 and Movies S1, S2, and S3.

*dsx* is expressed in ∼50 neurons in the brain and ∼310 neurons in the ventral nerve cord (VNC) of females [5, 10, 11, 12]. To specifically assess the role of brain *dsx*^+^ neurons in specifying female sexual behavior, we employed an intersectional approach, described in Figure 1A. We combined a *dsx*-specific Gal4 driver (*dsx*^*Gal4*^) [5] and a brain-specifically expressed flippase recombinase (*Otd-FLP*) [13] with a Gal4/FLP-responsive reporter or effector. These *dsx∩Otd* intersected females (*dsx*^*brain*^>*mGFP*) showed the typical *dsx* expression pattern in the brain but no expression in the VNC (Table S1; Figure 1B).

We assessed the behavioral effects of specifically activating brain *dsx*^+^ neurons by expressing the heat-activated ion channel TrpA1, which induces depolarization when the temperature is increased above 25°C [14]. We paired a *dsx*^*brain*^>*TrpA1* virgin female with a wild-type male at 22°C or 33°C (Figure 1C). At 22°C, *dsx*^*brain*^>*TrpA1* virgin females behaved normally, with males actively courting them (Figure 1D; Movie S1). Surprisingly, when thermally activated (at 33°C), *dsx*^*brain*^>*TrpA1* virgin females showed male-typical behaviors toward males (Figures 1E and S1; Movie S2). While wild-type *D. melanogaster* females never display male courtship behaviors, we found that *dsx*^*brain*^>*TrpA1* females spend ∼50% of their time courting males, performing the first steps of the behavioral ritual, such as following and tapping the target fly, as well as extending one or two wings (Figure S1). Activated females, however, did not engage in licking or abdominal bending associated with attempted copulation. Interestingly, most males targeted by the activated females showed greatly reduced levels of courtship and instead attempted to escape the female’s advances (Figures 1E and S1; Movie S2). *dsx*^*brain*^>*TrpA1* females were also attracted to wild-type females (Movie S3), showing no sex-specific bias in their behavioral response, as they spent a similar amount of time courting each sex in a preference assay (Figure 1F).

### Activation of *dsx*^+^ Neurons in the Female Brain Evokes Male-like Courtship Song

**Figure 2:**
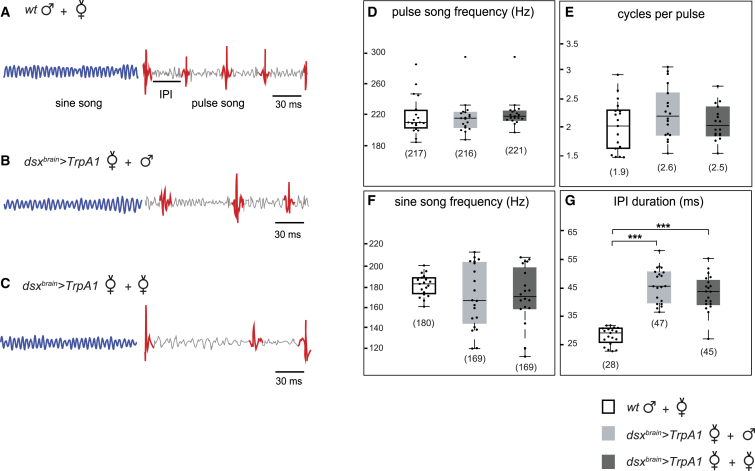
*dsx*^*+*^ Neurons in the Female Brain Evoke Pulse and Sine Song Courtship song was detected in recordings of 10 min. Sine song is shown in blue and pulses in red. (A) Close up of courtship song trace produced by a wild-type male courting a wild-type virgin female at 33°C. (B and C) Close up of courtship song traces produced by a *dsx*^*brain*^>*TrpA1* female paired with a wild-type male (B) or female (C) at 33°C. Timescale indicated in milliseconds (ms). (D–G) Boxplots of individual flies’ pulse song frequency in Hz (D), number of cycles per pulse (E), sine song frequency in Hz (F), and mean IPI in ms (G). Median with interquartile range is indicated by boxes. Mean values are indicated below each box. n = 20 flies recorded. Kruskal-Wallis ANOVA was performed for all assays (^∗∗∗^p < 0.0001). IPI, inter-pulse interval. See also Figure S2.

During courtship, *D. melanogaster* males vibrate one wing at a time producing a species-specific song that increases female receptivity [15, 16]. We thus tested whether *dsx*^*brain*^>*TrpA1* females are capable of producing male-like courtship song. Audio recordings from activated females paired with wild-type males or females showed characteristic acoustic features of male-like courtship song (shown in Figure 2A), including sine and pulse episodes (Figures 2B and 2C). How similar is activated female song to wild-type male song? The two have similar pulse and sine song frequency (Figures 2D and 2F) and number of cycles per pulse (Figure 2E). However, the inter-pulse interval (IPI), a species-specific parameter [16], was higher in activated female song than in wild-type male song (Figure 2G). We next quantified song events and examined bout structure in *dsx*^*brain*^>*TrpA1* females. We found that activated females sing less song than males; both the number of sine bouts per minute and the duration of sine bouts were significantly reduced when compared to males (Figures S2D and S2E). Although the number of pulse bouts per minute did not differ between activated females and wild-type males (Figure S2F), the duration of pulse bouts was significantly shorter in females (Figure S2G). Thus, *dsx*^*brain*^>*TrpA1* females display male-like pulse and sine song, albeit at much reduced levels compared to males. This is not surprising as male-specific *fru*^+^ and *dsx*^+^ neurons in the thorax contribute to song production [3, 17, 18]. In addition, a Dsx^M^-dependent sexually dimorphic muscle is required for the production of robust sine song [17]. Photoactivation of *fru*^+^ neurons in the thorax of headless females has previously been shown to elicit courtship song [19], leading to speculation that the motor program for song is present in females but lies dormant, because the neural commands in the brain required for song initiation are absent [3, 19]. In contrast, our data demonstrate that females possess brain neurons that, when activated, trigger song, as well as other male-like courtship behaviors.

### *dsx-*pC1 Neurons Induce Male-Typical Courtship Behaviors in Females

**Figure 3:**
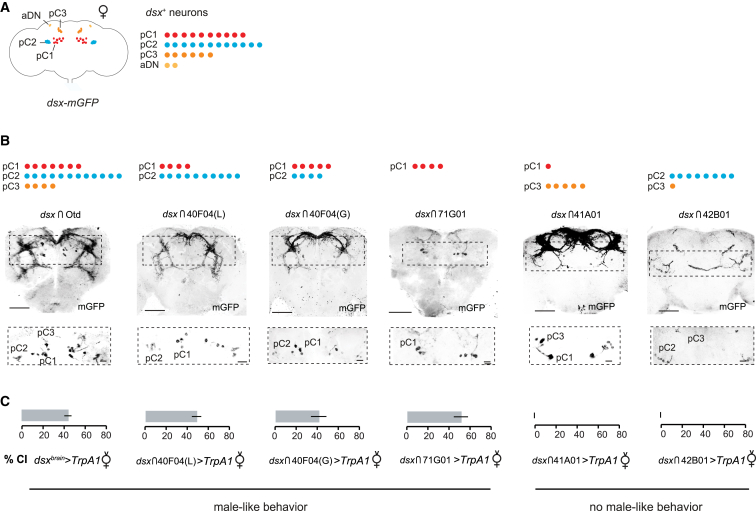
Mapping *dsx*^*+*^ Brain Neuronal Clusters Critical for Inducing Male-like Behaviors in Females (A) Schematic of *dsx*^*+*^ neuronal clusters in the female brain labeled by GFP in control females (*dsx*^*Gal4*^*/UAS-mGFP*). (B) Genetic subdivision of *dsx*^+^ neurons in the female brain combining different genetic tools (see Table S1) with *UAS*>*stop*>*mGFP*. mGFP expression is shown in black. The number of *dsx*^*+*^ neurons found in each brain cluster per hemisegment (shown in Table S1) is represented with dots. Scale bars, 50 μm. Higher magnification of the dorsal brain is shown in the bottom panel. Scale bars, 12.5 μm. (C) Behavioral effects of thermoactivating subsets of *dsx*^+^ neurons in the female brain. The graphs depict the courtship index (CI) displayed by intersected females in the presence of a wild-type male at 33°C. n = 12–15. Error bars, SD. No significant differences were observed between different intersected females displaying male-like courtship (p > 0.05; Mann-Whitney test). See also Figure S3.

*dsx*^+^ neurons in the female brain are distributed in discrete clusters: pC1, pC2, pC3 (also known as pCd [7]) and aDN [5, 10, 11, 12] (Figure 3A). As the female pC1 cluster is anatomically homologous to the male pC1 cluster [20], which includes the *fru*^+^ courtship promoting P1 neurons [3, 4, 21, 22], we reasoned that this cluster may be responsible for the activation of male-typical courtship behaviors in *dsx*^*brain*^>*TrpA1* females. To test this hypothesis, we exploited intersectional methods [5, 7, 8, 20, 23] to target distinct *dsx*^+^ neuronal clusters in the female brain (Figures 3A and 3B; Table S1). We first visualized intersected *dsx*^+^ neurons in the female and male nervous system (Figure 3B; Table S1). We next used these restricted lines to express TrpA1 to test whether activating distinct *dsx*^+^ clusters drives male-like courtship behaviors in females (Figure 3C). We found that females only displayed male-like courtship behaviors toward males in strains that intersected four or more pC1 neurons per hemisphere. In contrast, females in which the majority of pC2 neurons or pC3 neurons are artificially activated did not show male-like behaviors. The clearest role for pC1 neurons in inducing male-like behaviors was seen in *dsx∩71G01* females, where only four pC1 neurons are activated; these females showed high levels of courtship when paired with a wild-type male (∼50%; Figure 3C), displaying following, tapping, and wing extension behaviors (Figure S3A).

Activating the homologous neurons in *dsx∩71G01*>*TrpA1* males also triggered courtship behaviors (Figures S3B and S3C), consistent with previous findings [6, 20]. Unlike the corresponding females, these males display courtship behaviors in the presence or absence of a male or female, and at lower activating temperatures (30 versus 32°C; Figures S3J and S3K). Note that males have more pC1 neurons labeled by *dsx∩71G01* than females (∼17 versus approximately four neurons per brain hemisphere, respectively) (Table S1; Figures S3F–S3I), consistent with previous findings [7]. pC1 neurons in *dsx∩71G01* males include both *dsx*^+^/*fru*^−^ neurons and *fru*^+^/*dsx*^+^ P1 neurons (data not shown), while pC1 neurons in females are all *dsx*^+^/*fru*^−^ [7, 9, 10] and are thus distinct from the *fru*/*dsx*^+^ P1 subgroup in males. Thus, artificial activation of sexually dimorphic *dsx*-pC1 neurons promotes male courtship behaviors in both sexes.

### Volatile Compounds Induce Male Courtship Behaviors in Females

**Figure 4:**
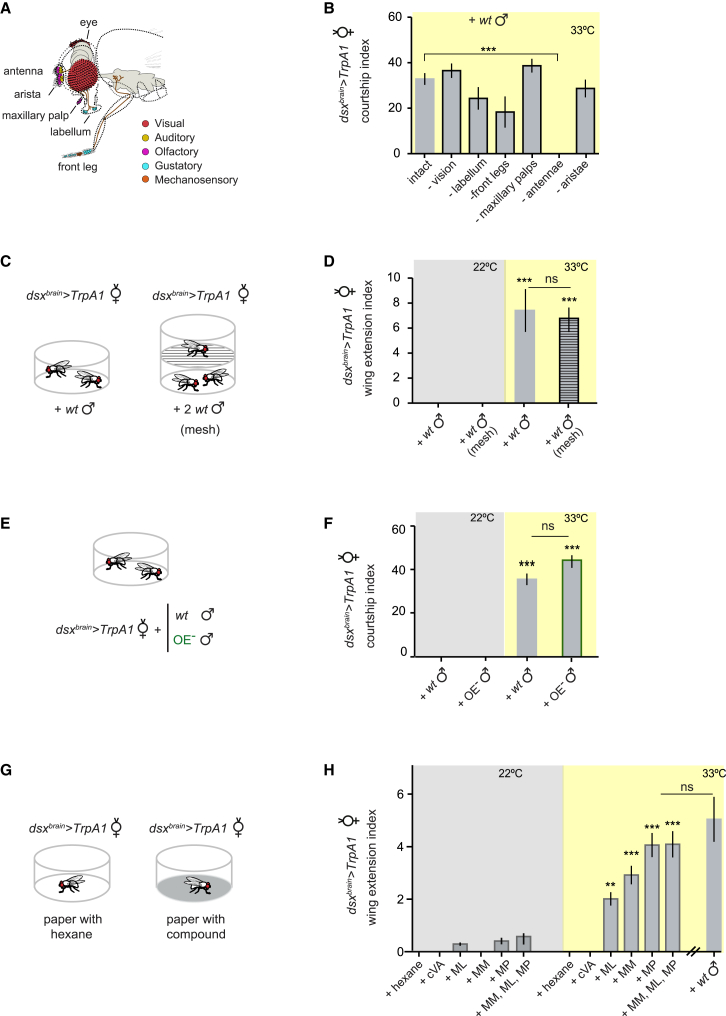
Identification of Pheromones Required for Inducing Male Courtship Behaviors in Females (A) Schematic showing appendages and associated sensory modalities involved in courtship behaviors (modified from [25]). (B) Courtship index displayed by *dsx*^*brain*^>*TrpA1* females with intact or severed sensory modalities when paired with a wild-type male at 33°C. n = 20–30. (C and D) Volatile olfactory cues trigger male-like behaviors in *dsx*^*brain*^>*TrpA1* females. (C) Schematic of assay in which a *dsx*^*brain*^>*TrpA1* female is paired either with a male in the same courtship chamber or separated from two males by mesh. (D) Courtship index displayed by *dsx*^*brain*^>*TrpA1* virgin females in contact or without contact (+ mesh) with male targets at 22°C (gray box) or 33°C (yellow box). n = 20. (E–H) Identification of pheromones promoting male-like behaviors in *dsx*^*brain*^>*TrpA1* females. (E) A *dsx*^*brain*^>*TrpA1* female was placed in a courtship chamber with either a male (*wt*) or an oenocyte-less male (OE^−^). (F) Courtship index displayed by *dsx*^*brain*^>*TrpA1* females paired with target flies at 22°C (gray box) or 33°C (yellow box). (G) Schematic of assay in which a *dsx*^*brain*^>*TrpA1* female is placed in a courtship chamber containing a filter paper soaked with either hexane (solvent), 11-cis-Vaccenyl acetate (cVA), methyl laurate (ML), methyl myristate (MM), methyl palmitate (MP) or a ML, MM, and MP mix. (H) Wing extension index displayed by a solitary *dsx*^*brain*^>*TrpA1* virgin female in the presence of each compound at 22°C (gray box) or 33°C (yellow box). (n = 20). Statistical comparisons of the indicated genotypes at 33°C were made against genotypes at 22°C unless otherwise indicated. A Kruskal-Wallis ANOVA test was performed in (B), (D), (F), and (H) (^∗∗^p < 0.001; ^∗∗∗^p < 0.0001). Error bars, SEM; ns, not significant. Courtship behaviors were measured over 3 min. See also Figure S4.

In contrast to *dsx*^*brain*^>*TrpA1* males, *dsx*^*brain*^>*TrpA1* females do not show courtship behaviors at 33°C in isolation (data not shown), suggesting that activated females require additional sensory cues provided by another fly to trigger courtship behaviors. To identify such sensory cues, we disrupted individual sensory modalities known to stimulate courtship behavior in males, in *dsx*^*brain*^>*TrpA1* females [2, 24] (Figure 4A). To block vision in general, we paired *dsx*^*brain*^>*TrpA1* females with headless males in the dark. To block gustation, we removed the females’ labella or front legs. To block audition, we removed the females’ aristae. In all of these cases, *dsx*^*brain*^>*TrpA1* females still showed male-like courtship behaviors (Figure 4B). In contrast, when we blocked olfaction by removing the antennae, the *dsx*^*brain*^>*TrpA1* females did not court, suggesting that olfactory cues contribute to the induction of male-like behaviors (Figure 4B). Moreover, when we placed *dsx*^*brain*^>*TrpA1* females in close proximity to a pair of males, without allowing physical contact, the activated females showed courtship-specific wing extension (Figures 4C and 4D), further indicating that male-like courtship in *dsx*^*brain*^>*TrpA1* females can be induced by volatile olfactory cues. Interestingly, removing the maxillary palps did not block courtship behavior (Figure 4B), suggesting that the relevant olfactory cues are detected by the antennae, not the maxillary palps.

### Methyl Laurate, Methyl Myristate, and Methyl Palmitate Are Stimulatory Pheromones for Male-like Behaviors in Females

The finding that *dsx*^*brain*^>*TrpA1* females court both males and females (Figure 1F) suggested male-like behaviors might be induced by pheromones common to both sexes. Oenocyte cells (OEs) are the main sites of pheromone production in *Drosophila*, as ablation of these cells eliminates cuticular hydrocarbons, which act as pheromones [24, 26]. Surprisingly, males with and without OE cells elicited similar courtship levels in activated females (Figures 4E and 4F), suggesting that the pheromones that stimulate courtship in activated females are not produced by oenocytes. The only known volatile pheromone found in OE-less males is 11-cis-Vaccenyl acetate (cVA), as it is produced in the male ejaculatory bulb [27, 28]. However, cVA is missing in virgin females [29]; thus, it cannot explain why activated *dsx*^*brain*^>*TrpA1* females are stimulated to court virgin females. Indeed, cVA did not induce wing extension in solitary *dsx*^*brain*^>*TrpA1* females at 33°C (Figures 4G and 4H).

It was recently shown that methyl laurate (ML), methyl myristate (MM), and methyl palmitate (MP) are cuticular odorants that elicit short-range attraction behavior in both sexes and are present approximately equally in males and females [30]. Interestingly, ML, MM, and MP are found at normal levels in oenocyte-less flies as well as in other *Drosophila* species [30]. We tested whether exposing solitary *dsx*^*brain*^>*TrpA1* females to candidate odors promoted wing extension at 33°C. Whereas hexane, the solvent used to dilute the compounds, did not elicit wing extension, ML, MM, or MP all triggered this behavior (Figures 4G and 4H). In particular, MP triggered the highest levels of wing extension in activated females in our assay, which were similar to the levels elicited by the three compounds together (Figure 4H). These cuticular compounds appear to act at short distances in *dsx*^*brain*^>*TrpA1* females, as these females are generally in close contact with their courtship targets, and show decreased courtship levels in bigger chambers (<29% versus >43% courtship index, n = 25, p < 0.05).

We next asked whether *dsx*^*brain*^>*TrpA1* females could be stimulated by other *Drosophila* species [31, 32], which are not normally courted by *D. melanogaster* males [33]. We found that ∼60% of activated *dsx*^*brain*^>*TrpA1* females courted flies of related species within the *melanogaster subgroup* (*D. simulans* and *D. yakuba*), and more distantly related species within the subgenus *Sophophora* (*D. wilistoni*) (Figures S4A and S4B). Only ∼20%–30% of activated females showed courtship toward *D. virilis* flies, which are distantly related to *D. melanogaster*, having diverged about 60 million years ago [32]. While *D. melanogaster*, *D. simulans*, *and D. yakuba* produce either MP and/or ML and MM [30], we found that none of these methyl pheromones are present in *D. virilis* flies (Figure S4C). Interestingly, coating *D. virilis* males with MP increased both the number of *dsx*^*brain*^>*TrpA1* females displaying male courtship behaviors (∼56%) and the courtship levels when compared to hexane-treated *D. virilis* flies (Figure S4D). Our data support a role for MP as an important stimulatory olfactory cue for male courtship behaviors in *dsx*^*brain*^>*TrpA1* females.

### Sexually Dimorphic Usage of Common Circuitry

If females have neurons capable of inducing courtship, why do they not normally display these male behaviors? Two observations may be instructive: first, females require more stimulation—both sensory and thermogenetic—to show male courtship behavior. *dsx∩71G01*>*dTrpA1* females require higher temperatures than males, in addition to olfactory cues, to show activation-induced courtship (Figures S3A–S3C, S3J, and S3K). Second, stimulation of pC1 neurons in *dsx∩71G01*>*dTrpA1* females induces different behaviors depending on the level of stimulation, which presumably correlates with heat-induced changes in the neural activity of pC1 neurons [14, 34, 35]). We found that raising the temperature from 22°C to 27°C increases female receptivity, seen in their latency to copulation (670 ± 50 s versus 1,452 ± 175 s, n = 20, p < 0.05) and their ability to elicit higher levels of male courtship (∼90% versus ∼66% courtship index, n = 20, p < 0.001). This is consistent with a previous study showing pC1 neurons are responsive to courtship-relevant stimuli, such as cVA and courtship song, and promote receptivity when activated at low temperatures [7]. We further found that raising the temperature from 27°C to 33°C causes females to transition from female-typical behaviors to male-typical behaviors (Figure S3L).

The reason males court, while females do not, may be due to an intrinsic difference in this key node in the courtship circuit. This could be a consequence of (1) differences in the number of pC1 neurons (e.g., fewer neurons in females are not sufficient to trigger male courtship behaviors), (2) differences in the intrinsic properties of pC1 neurons (e.g., they are less excitable in females), and/or (3) differences in the connectivity of pC1 neurons (e.g., they receive more inhibition or less excitation in females). Discrete pC1 neurons may function as a switch for courtship versus aggression in males, with *fru*^+^*/dsx*^+^ neurons mediating courtship and *fru*^−^/*dsx*^+^ mediating aggression [22]. Thus, the pC1 node in both sexes appears to integrate sensory cues to regulate socio-sexual behavior (courtship and aggression in males [3, 4, 21, 22, 36], receptivity in females [7]), but when it is strongly activated outside the physiological range in females, it can activate downstream circuitry (e.g., *fru*^+^ thoracic neurons [3, 19]) for courtship that normally lies dormant.

We have identified MP and related cuticular pheromones as stimulators of male-like behaviors in females. Interestingly, MP also triggers male courtship behaviors in solitary activated males (Figures S3D and S3E). ML, MM, and MP induce attraction behavior through Or88a olfactory neurons in the antenna, which can facilitate mate finding [30]. In addition, ML stimulates male copulation behavior via Or47b olfactory neurons [30, 37]. Thus, Or88a and Or47b-expressing neurons are strong candidates for mediating male courtship behaviors in *dsx*^*brain*^>*TrpA1* females.

How are pC1 activation and MP stimulation integrated? This cuticular pheromone might modulate the activity of pC1 neurons or, alternatively, pheromone-responsive circuits and pC1 neurons might converge on downstream neurons that mediate courtship behaviors in females and males. It should be noted that, since pC1 neurons are probably maximally activated at 33°C in our experiments (Figures S3J and S3K), MP is unlikely to further activate these neurons. This suggests that pC1 activation and MP stimulation may be integrated in downstream neurons.

It was previously proposed that male courtship behavior, such as song, may result from the activation of brain neurons unique to males [3, 19]. Here, we show that the female brain is capable of inducing male-like behaviors. Notably, a male-sexual behavior effector circuit has been shown to be present in the brain of female mice, which is normally repressed [38]. Hence, the existence of functional neuronal circuitry underlying male-specific behaviors in the normal female brain seems to be a conserved feature of courtship circuit organization [39].

## Author Contributions

C.R., S.P., B.B., and S.F.G. designed the experiments and contributed to data analysis, interpretation, and discussion. C.R., S.P., H.J.P., T.N., B.B., L.A.D.D., and H.K.M.D. conducted the experiments. The manuscript was written by C.R. and corrected by S.F.G. All authors contributed to the revision of the final manuscript.

## Supplemental Information

Document S1. Supplemental Experimental Procedures, Figures S1–S4, and Table S1

Movie S1. *dsx*^*brain*^*>TrpA1* Females Are Normally Courted by a Wild-Type Male at 22°C, Related to Figure 1 Movie showing a wild-type male displaying normal courtship behaviors towards a *dsx*^*brain*^*>TrpA1* female at the control temperature (22°C).

Movie S2. Activation of *dsx*^+^ Neurons in Females Induces Male-like Courtship Behaviors toward a Wild-Type Male, Related to Figure 1 Movie showing a *dsx*^*brain*^*>TrpA1* virgin female displaying male-typical courtship behaviors, such as following and wing extension, directed towards a wild-type male when thermally activated (at 33°C).

Movie S3. Activation of *dsx*^+^ Neurons in Females Induces Male-like Courtship Behaviors toward a Wild-Type Female, Related to Figure 1 Movie showing a *dsx*^*brain*^*>TrpA1* virgin female displaying male-typical courtship behaviors, such as following and wing extension, directed towards a wild-type female when thermally activated (at 33°C).

Document S2. Article plus Supplemental Information
