# Supplementary material for: Activation of Latent Courtship Circuitry in the Brain of Drosophila Females Induces Male-like Behaviors
Source: Curr Biol. 2016 Sep 26;26(18):2508–15. doi: 10.1016/j.cub.2016.07.021 (PMC5049544; doi:10.1016/j.cub.2016.07.021)
Supplement: Document S1. Supplemental Experimental Procedures, Figures S1–S4, and Table S1 [file mmc1.pdf]

Current Biology, Volume *26*

Supplemental Information

Activation of Latent Courtship Circuitry in the Brain of *Drosophila* Females Induces Male-like Behaviors

Carolina Rezával, Siddharth Pattnaik, Hania J. Pavlou, Tetsuya Nojima, Birgit Brüggemeier, Luis A.D. D'Souza, Hany K.M. Dweck, and Stephen F. Goodwin

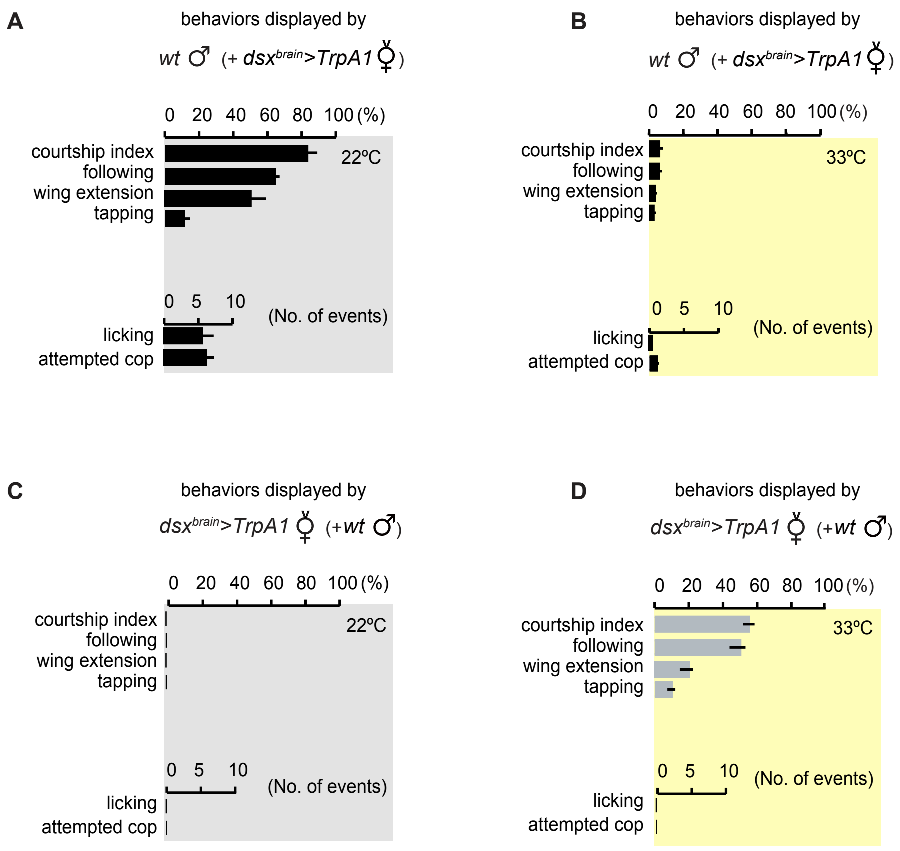

Figure S1, related to Figure 1. Behavioral characterization of *dsx^{brain}>TrpA1* females and wild-type males. Courtship index displayed by wild-type (*wt*) males paired with *Otd-FLP/UAS>stop>TrpA1;dsx^{Gal4}*/+ virgin females (indicated as *dsx^{brain}>TrpA1*) at 22°C (grey panel; **A**) or 33°C (yellow panel; **B**). Courtship index displayed by *dsx^{brain}>TrpA1* virgin females paired with *wt* males at 22°C (grey panel; **C**) or 33°C (yellow panel; **D**). Flies were recorded at high magnification to monitor individual courtship steps. For following, wing extension and tapping the total time engaged in these steps was recorded; for licking and attempted copulation (attempted cop), it was the total number of events. n=15. Error bars indicate SEM.

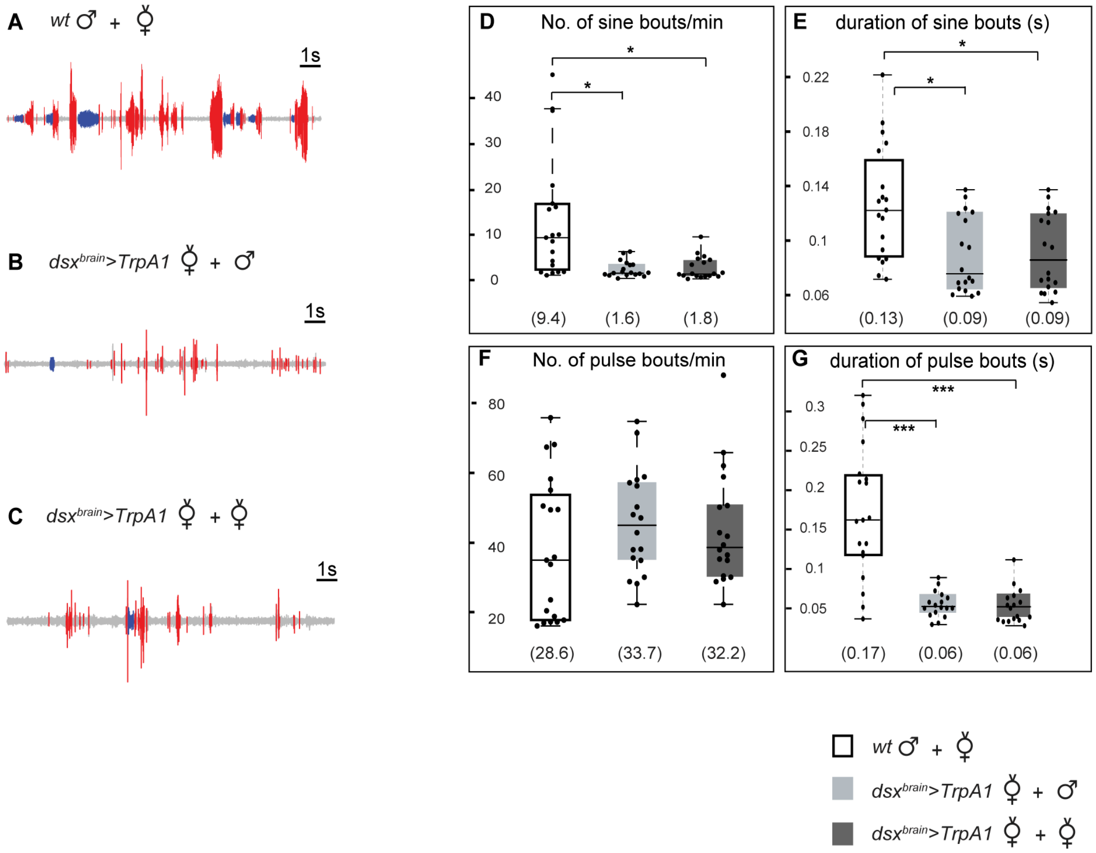

Figure S2, related to Figure 2. Characterization of song events and bout structure in *dsx^{brain}>TrpA1* females. (**A**-**C**) Courtship song was detected [S1] in recordings of 10 min. Sine song is shown in blue and pulses in red. (**A**) Overview of courtship song trace produced by a wild-type male courting a wild-type virgin female at 33°C. (**B**,**C**) Overview of courtship song traces produced by a *dsx^{brain}>TrpA1* female paired with a wild-type male (**B**) or female (**C**) at 33°C. Timescale indicated in seconds (s). (**D**-**G**) Box plots of No of sine bouts/minute (**D**), duration of sine bouts (s) (**E**), No of pulse bouts/minute (**F**) and duration of pulse bouts (s) (**G**). Median with interquartile range is indicated by boxes. Mean values are indicated below each box. n= 19-20 flies recorded. Kruskal-Wallis ANOVA was performed in all assays (*p < 0.01; ***p < 0.0001).

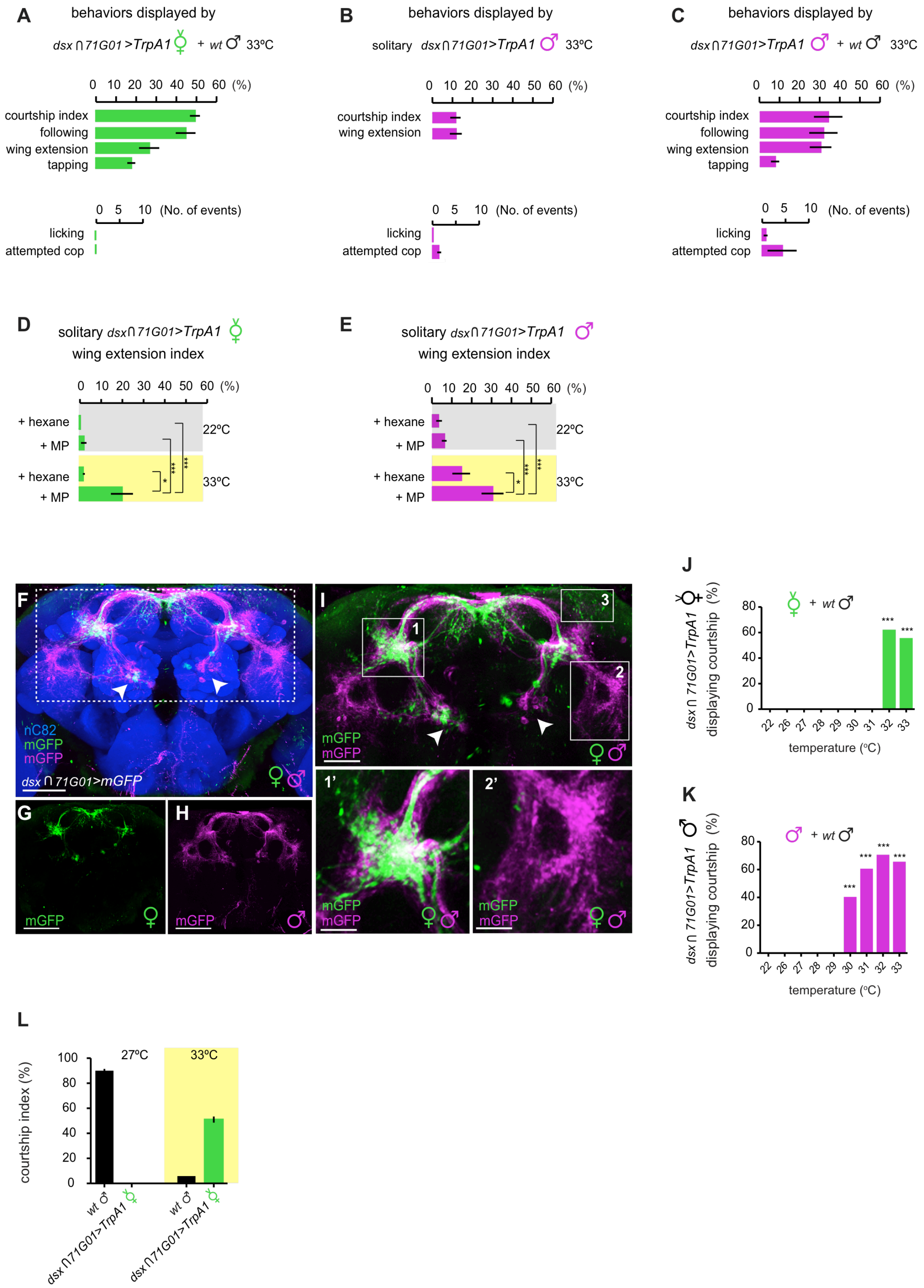
A
behaviors displayed by
dsx ∩ 71G01 >TrpA1 ⚥ + wt ♂ 33ºC
courtship index
following
wing extension
tapping
licking
attempted cop
B
behaviors displayed by
solitary dsx∩71G01>TrpA1 ♂ 33ºC
courtship index
wing extension
licking
attempted cop
C
behaviors displayed by
dsx∩71G01>TrpA1 ♂ + wt ♂ 33ºC
courtship index
following
wing extension
tapping
licking
attempted cop
D
solitary dsx∩71G01>TrpA1 ⚥
wing extension index
+ hexane
+ MP
22ºC
33ºC
E
solitary dsx∩71G01>TrpA1 ♂
wing extension index
F
nC82
mGFP
mGFP
dsx ∩ 71G01>mGFP
G
H
I
J
dsx ∩ 71G01>TrpA1 displaying courtship (%)
temperature (ºC)
K
L
courtship index (%)
27ºC
33ºC
wt ♂
dsx ∩71G01>TrpA1

Figure S3, related to Figure 3. Anatomical and behavioral characterization of *dsx*-pC1 neurons in females and males. (**A**-**C**) Behavioral assays were recorded at higher magnification to monitor individual courtship steps displayed either by *dsx∩71G01>TrpA1* virgin females paired with wild-type males (**A**), *dsx∩71G01 >TrpA1* males paired with wild-type males (**B**) or solitary *dsx∩71G01>TrpA1* males at 33°C (**C**). For following, wing extension and tapping the total time engaged in these steps was recorded; for licking and attempted copulation (attempted cop), it was the total number of events. n=12-15. Error bars indicate SEM. Genotype: *GMR71G01-lexA/UAS>stop>TrpA1;LexAop2-FLP/*dsx^{Gal4}. (**D**-**E**) Effects of methyl palmitate (MP) on courtship behavior. Wing extension index displayed by a solitary *dsx∩71G01>TrpA1* virgin female (**D**) or male (**E**) in the presence of hexane or MP at 22°C (grey box) or 33°C (yellow box). n=20. A Kruskal-Wallis ANOVA test was performed (*p < 0.05; ***p < 0.0001). Error bars indicate SEM. Wing extension index were measured over 3 minutes. (**F**-**I**) *dsx*-pC1 neurons are sexually dimorphic. (**F**) Confocal images of the female (**G**) and male (**H**) brains of *dsx∩71G01>mGFP* flies were registered onto an intersex template brain for *D. melanogaster* generated by the Jefferis lab [S2-S4] (https://zenodo.org/record/10591). mGFP staining in the female is shown in green (**F, G**) and mGFP staining in the male is shown in magenta (**F**,**H**). nc82 staining is shown in blue (**F**). Arrowheads indicate cell bodies. Scale bars: 25 µm (**F**) and 50 µm (**G**,**H**). Genotype: *GMR71G01-lexA/UAS>stop>myr::GFP;LexAop2-FLP/*dsx^{Gal4} (indicated as *dsx∩71G01>mGFP*). (**I**) Higher magnification of the dorsal brain shown in **F**. Arrowheads indicate cell bodies. Note that *dsx*-pC1 neurons show common arborizations in the brain of males and females (*e.g.,* region box 1), as well as male-specific innervations (*e.g.,* region box 2) and female-specific innervations (*e.g.,* region box 3). Scale bar: 25 µm. Higher magnification of region box 1 and 2 are shown in the lower panels (1' and 2'). Scale bars: 12,5 µm. (**J**-**K**) Characterization of the minimal temperature required in *dsx∩71G01>TrpA1* females (**J**) and males (**K**) to display courtship behaviors in the presence of a wild-type male. The graphs depict percentage of flies displaying courtship at increasing temperatures. Statistical comparisons of the indicated genotypes at different temperatures were made against genotypes at 22°C (Fisher exact Test; ***p < 0.0001). n=15-20. (**L**) Courtship assays for *dsx∩71G01>TrpA1* virgin females paired with wild-type males at 27°C or 33°C. The courtship indices displayed by both males and females are reported. The courtship levels displayed by *wt* males at 33°C are significantly different from those at 27°C, and the courtship levels displayed by *dsx∩71G01>TrpA1* females at 33°C are significantly different from those at 27°C (Kruskal-Wallis ANOVA; p< 0.0001). *UAS>stop>TrpA1/+;* dsx^{Gal4}/+ virgin females and *LexAop-FLP/+;71G01-LexA/+* virgin females did not display male-like courtship at 27°C or 33°C (data not shown). No significant differences in the courtship levels displayed by *wt* males paired with either *UAS>stop>TrpA1/+;* dsx^{Gal4}/+ control virgin females or *LexAop-FLP/+;71G01-LexA/+* control virgin females were found between 27°C and 33°C (data not shown). n=15-22. Error bars indicate SEM.

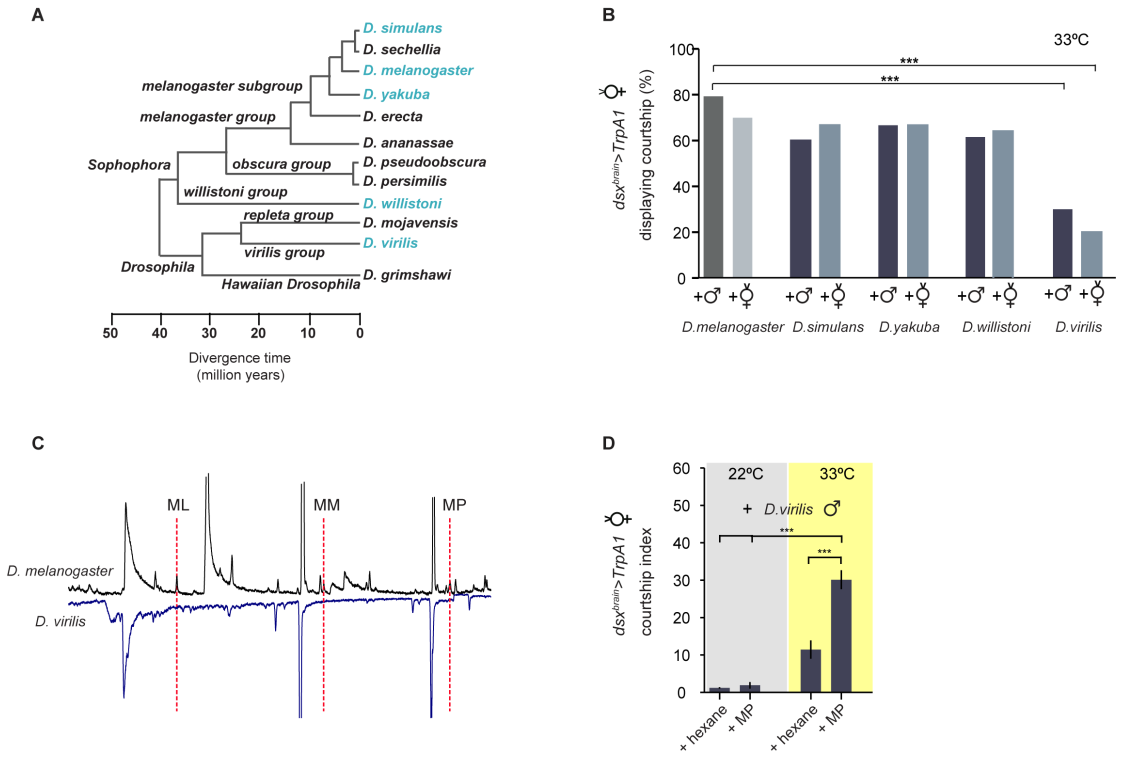

Figure S4, related to Figure 4. Activation of female brain *dsx*$^+$ neurons triggers male-courtship behaviors towards other *Drosophila* species.

(**A**) Schematic depicting phylogenetic relationships and estimated divergence times among species in the genus *Drosophila*. Modified from http://arthropods.eugenes.org/DroSpeGe/. (**B**) Percentage of *D. melanogaster Otd-FLP/UAS>stop>TrpA1;dsx*^{Gal4}*/+* virgin females (indicated as *dsx*^{brain}*>TrpA1*) displaying male-like courtship behaviors when paired with other *Drosophila* species at 33°C. A Fisher exact Test was performed (***$p < 0.0001$). n=15-20. (**C**) Representative TDU-GC-MS traces of individual *D. melanogaster* and *D. virilis* males. Methyl laurate (ML), methyl myristate (MM) and methyl palmitate (MP) are shown. None of these methyl pheromones are present in D. virilis males or females (data not shown). **(D)** Courtship index displayed by *dsx*^{brain}*>TrpA1* virgin females in the presence of *D.virilis* males perfumed with either hexane or methyl palmitate (MP) at 22°C or 33°C. Kruskal-Wallis ANOVA was performed (*** $p < 0.0001$). n=20-30. Error bars indicate SEM.

Intersection	Genotype	Number of *dsx*$^{+}$ neurons in females				
		**pC1**	**pC2**	**pC3**	**aDN**	**Abg**
dsx	*dsx*^{Gal4}*/UAS-mGFP*	10.6 ± 0.4	12.8 ± 0.5	6.6 ± 0.1	1.7 ± 0.3	311.3 ± 4.2
dsx∩Otd	*Otd-FLP/UAS>stop>myr::GFP; dsx*^{Gal4}*/+*	7.8 ± 0.2	11.6 ± 0.2	4.4 ± 0.3	0 ± 0	2.3 ± 2.4
dsx∩40F04(L)	*54785 (GMR40F04-lexA)/UAS>stop>myr::GFP; LexAop2-FLP/dsx*^{Gal4}	4.9 ± 1.1	10.6 ± 2.0	0 ± 0	0 ± 0	35.3 ± 1.2
dsx∩ 40F04(G)	*50094(GMR40F04-Gal4)/UAS>stop>myr::GFP; dsx*^{FLP}*/+*	5.1 ± 1.0	4.4 ± 1.3	0 ± 0	0 ± 0	2.6 ± 2.4
dsx∩71G01	*54733 (GMR71G01-lexA)/UAS>stop>myr::GFP; LexAop2-FLP /dsx*^{Gal4}	4.4 ± 0.8	0 ± 0	0 ± 0	0 ± 0	26.5 ± 2.5
dsx∩41A01	*54787 (GMR41A01-LexA)/UAS>stop>myr::GFP; LexAop2-FLP/dsx*^{Gal4}	1.2 ± 0.4	0 ± 0	5.7 ± 0.9	0 ± 0	13.3 ± 3
dsx∩42B01	*42B01-Gal4/ UAS>stop>myr::GFP; dsxFLP/+*	0 ± 0	8.4 ± 2.9	1.2 ± 0.4	0 ± 0	0 ± 0

Intersection	Genotype	Number of dsx^+ neurons in males							
		**pC1**	**pC2**	**pC3**	**aDN**	**SN**	**TN1**	**TN2**	**Abg**
dsx	*dsx^{Gal4}/UAS-mGFP*	52.5 ± 3.5	61.8 ± 4.6	12.8 ± 1.4	1.6 ± 0.8	1.7 ± 0.8	6.5 ± 0.5	22.2 ± 0.7	262.4 ± 16.9
dsx∩Otd	*Otd-FLP/UAS>stop>myr::GFP; dsx^{Gal4}/+*	46.2 ± 7.2	56.2 ± 7.6	11.4 ± 1.1	1.7 ± 0.8	0 ± 0	0 ± 0	0 ± 0	0 ± 0
dsx∩40F04(L)	*54785 (GMR40F04-lexA)/UAS>stop>myr::GFP; LexAop2-FLP/dsx^{Gal4}*	19.4 ± 3.1	32.3 ± 6.9	0.03 ± 0.17	0 ± 0	0 ± 0	6.4 ± 3.0	0.4 ± 0.7	34.1 ± 6.1
dsx∩ 40F04(G)	*50094 (GMR40F04-Gal4)/UAS>stop>myr::GFP; dsx^{FLP}/+*	2.9 ± 1.1	4.7 ± 1.6	0 ± 0	0 ± 0	0 ± 0	1.6 ± 0.8	0 ± 0	6.1 ± 1.4
dsx∩71G01	*54733 (GMR71G01-lexA)/UAS>stop>myr::GFP; LexAop2-FLP /dsx^{Gal4}*	17.2 ± 1.6	0.9 ± 1.3	0 ± 0	0 ± 0	0 ± 0	5.2 ± 0.8	0 ± 0	15.5 ± 4.8
dsx∩41A01	*54787 (GMR41A01-LexA)/UAS>stop>myr::GFP; LexAop2-FLP/dsx^{Gal4}*	6.0 ± 2.9	5.3 ± 4.6	2.7 ± 2.9	0 ± 0	0 ± 0	7.6 ± 1.9	0.2 ± 0.4	12.2 ± 5
dsx∩42B01	*42B01-Gal4/ UAS>stop>myr::GFP; dsxFLP/+*	0 ± 0	4.5 ± 1.8	1 ± 0	0 ± 0	0 ± 0	0 ± 0	0 ± 0	0 ± 0

Table S1, related to Figure 3. Quantification of intersected *dsx^+* neurons in the central nervous system.

Quantification of *dsx^+* intersected neurons targeted using *dsx* enhancer-LexA or Gal4 lines. Counts represent the number of intersected *dsx^+* neurons for each of the indicated clusters in the female and

male brain (per hemisegment) and ventral nerve cord, including the abdominal ganglion (Abg). Mean ± standard deviation is shown. Counts of *dsx*–positive neurons were obtained by annotating and counting each individually stained cell with anti-GFP through a series of optical sections within a stack. n= 12-15.

Supplemental Experimental Procedures

Fly stocks

All flies were raised on standard medium in a 12 hr light/12 hr dark cycle at 22°C or 25°C on standard medium at 50% relative humidity. Wild-type flies were obtained from a *Canton-S* (CS) strain. *UAS-StingerII, UAS-mCD8::GFP, UAS-hid* [S5], *tubP-Gal80*^{ts} [S6], *LexAop2-FLP, 54785* (*GMR40F04-lexA*), *54733* (*GMR71G01-lexA*), *54787* (*GMR41A01-LexA*)*, 50094-Gal4* (*GMR40F04-Gal4*) and *42B01-Gal4* [S7, S8] were obtained from the Bloomington stock centre. Other fly stocks used include *dsx*^{Gal4} [S9], *dsx*^{FLP} [S10], *Otd-nls:FLPo* [S11] (provided by D.J. Anderson, Caltech), *PromE(800)-Gal4* [12] (provided by J. Levine, University of Toronto), *D. virilis, D.willistoni, D.simulans* and *D.yakuba* (provided by M.J. Ritchie, University of St Andrews), *UAS>stop>myr::GFP* [S13] (provided by G. Rubin, Janelia Farm), *fru*^{Gal4} [S14], *UAS>stop>mCD8::GFP* [S15] and *UAS>stop>trpA1*^{myc} [S16] (provided by B. Dickson; Janelia Farm).

Immunohistochemistry

Flies were reared at 25°C and aged for 4–6 days prior to dissection and staining. Samples were fixed in 4 % paraformaldehyde in Phosphate Buffered Saline (PBS) for 20 minutes at room temperature, then washed three times for 15 mins in PBT (1 x PBS, 0.4 % Triton X-100), and incubated for 40 min in PTN (PBT, 5% normal goat serum; Scottish Diagnostics and Molecular Probes). The specimens were incubated at 4°C for 4-5 days in PTN containing the primary antibodies. Next, they were washed in PBT for 2-4 hr and incubated in PTN containing the appropriate secondary antibodies for 2 days at 4°C. They were washed in PBT for 2-4 hr. Stained specimens were mounted in VectaShield (Vector Lab) on Polylysine treated microscope slides (BDH). Primary antibodies used were: rabbit anti-GFP (1:1000, Invitrogen Molecular Probes, Carlsbad, CA), chicken anti-GFP (1:1000, Abcam), mouse mAb nC82 (1:10, DSHB, Univ. of Iowa, IA). Secondary antibodies used were: anti-rabbit Alexa Fluor488, and anti-mouse Alexa Fluor546 conjugates (1:300 Invitrogen Molecular Probes, Carlsbad, CA) and anti-chicken Alexa Fluor488 (1:500 Invitrogen Molecular Probes, Carlsbad, CA). Confocal stacks were taken with Olympus FV1000 and Leica SP5 microscopes. Images were processed in Amira 5.2 (Mercury Systems).

Brain image registration

Confocal images of the female and male brain of *GMR71G01-lexA)/UAS>stop>myr::GFP; LexAop2-FLP/dsx*^{Gal4} (*dsx∩R71G01)* were registered onto an intersex template brain for *D. melanogaster*

generated by the Jefferis lab, using a Fiji graphical user interface (GUI), as previously described [S2-S4] (https://zenodo.org/record/10591). Four male and female brain samples were analysed.

Behavioral assays

Flies were kept in vials in groups of 2 for 6–8 days before behavioral assays at 22°C or 25°C. Single-pair courtship tests [S17] were performed in round chambers (10 mm diameter × 4 mm height or 19 mm diameter × 4 mm height -for experiments in bigger chambers-) and recorded at different temperatures. Courtship index was measured as the proportion of time during 10 min that the female or male spent exhibiting any male-typical courtship behaviors (*e.g.,* following, wing vibration, tapping, licking, attempted copulation) upon courtship initiation. For tapping, following and wing extension, the total time engaged in these steps during 10 min was recorded; for licking and attempted copulation, it was the total number of events. For the courtship preference assay: a single dsx^{brain}>*TrpA1* female was placed in a chamber (19 mm diameter × 4 mm height) with a wild-type male and a wild-type female at 33°C. The proportion of time during 10 min that the dsx^{brain}>*TrpA1* female spent exhibiting any male-typical courtship behaviors towards the male or female was measured. In Figure 4H, Figure S3D-E and Figure S4D, courtship index was measured within 3 min to avoid evaporation of the candidate odors. In order to compare different behavioral assays, we also quantified courtship index and wing vibration index over 3 min upon courtship initiation in in Figure 4 B,D,F. For the experiments involving severed sensory modalities, tarsi, antennae, and maxillary palps were surgically removed bilaterally under anesthesia 1–3 days prior to behavioral testing, as per [S18]. The labellum or aristae were surgically removed under anesthesia 2–3 hr prior to behavioral testing, as per [S18]. To eliminate the contribution of visual cues, behavioral tests were carried out under far-red light. For cuticular compounds assays in Figure 4H or S3D-E, candidate compounds were diluted in hexane, applied onto a whatman filter paper and left for 5 minutes at 33°C to allow the solvent to evaporate. Methyl palmitate (Sigma) and methyl myristate (Sigma) were used at 1:10 (as per [S19]), whereas methyl laurate (Sigma) was tested at 1:1000. Ten µl of concentrated cVA (Cayman chemicals) was tested. Solitary females were behaviorally assessed in courtship chambers containing the filter paper with either hexane or candidate compounds at 22°C or 33°C. For the oenocyte-less experiments, adults lacking oenocytes were obtained from the progeny of the cross of +*: PromE(800)-Gal4, tubP-Gal80ts;+ to +: UAS-StingerII, UAS-hid/CyO;*+, following the protocol published in [S12]. For perfuming experiments (Figure S4D), 10µl of hexane was pippeted into a glass tube. After the solvent was evaporated, 10 flies were transferred to the glass tube and subjected to gentle pulses for a total of 5 minutes. The treated flies were then transferred to fresh food vials to recover and then tested for behavior. For experiments involving MP, after the initial procedure with hexane, the flies were transferred to a glass tube with MP (1:10 in hexane) and vortexed them for 1 minute before the behavioral assays. For experiments in Figure S3L, *dsx∩71G01>TrpA1(LexAopFlp/UAS>stop>TrpA1;*dsx^{Gal4}*/71G01-LexA)*, *UAS>stop>TrpA1/+;* dsx^{Gal4}/+ and *LexAop-FLP/+; dsx∩71G01-LexA/*+ virgin females paired with *wt* males were tested at 27°C or 33°C. In addition, to test the transition of behaviors upon increasing the

temperature the same *dsx∩71G01>TrpA1* females and *wt* males tested at 27°C were then evaluated at 33°C (data not shown).

TD-GC-MS.

Individual flies were placed in standard microvials in thermal desorption tubes and transferred using a GERSTEL MPS 2 XL multipurpose sampler into a GERSTEL thermal desorption unit (www.gerstel.de). After desorption at 200 °C for 5 min with solvent venting, the analytes were trapped in the liner of a GERSTEL CIS 4 Cooled Injection System at−50 °C, using liquid nitrogen for cooling. The components were transferred to the GC column by heating the programmable temperature vaporizer injector at 12 °C/s up to 210 °C and then held for 5 min. The GC-MS device (Agilent GC 7890A fitted with an MS 5975C inert XL MSD unit; www.agilent.com) was equipped with an HP5-MS UI column (19091S-433UI; Agilent Technologies) and operated as follows. The temperature of the gas chromatograph oven was held at 40 °C for 3 min and then increased by 5 grd/min to 200 °C and then by 20 grd/min to 260 °C, with the final temperature held for 15 min. For MS, the transfer line was held at 260 °C, the source was held at 230 °C, and the quad was held at 150 °C. Mass spectra were taken in EI mode (at 70 eV) in the range from 33 m/z to 500 m/z. The structures of most of the cuticular compounds were confirmed by comparison with reference compounds measured at the same conditions.

Song assays

For recording song, experimental flies were paired with a wild-type male or female in cylindrical courtship chambers with a diameter of 10 mm and a height of 4 mm. Sound was recorded with a CMP-5247TF-K microphone in an Insectavox [S20], that was modified by adding two peltier elements to its sides, that allow raising ambient temperature in the box to 33°C. Temperature was controlled with a National Instruments DAQ. Recordings were analyzed using the MATLAB toolbox FlySongSegmenter [S1], and additional custom-made MATLAB software (available upon request). All *dsx*^{brain}*>TrpA1* recordings were examined to ensure only females were singing. For pulse analysis we used the default settings of FlySongSegmenter. For sine analysis we considered sine trains exceeding a threshold of 0.2 of normalised amplitude (to exclude background noise).

Statistics

Behavioral means were compared using Kruskal-Wallis ANOVA or Mann-Whitney test and Dunn’s post hoc statistical test were indicated. For Fisher's exact test, two-tail *p* values were compared with controls. Statistical analyses were performed with GraphPad Prism software (version 6.0b, SPSS Inc.).

Supplemental References

S1. Arthur, B.J., Sunayama-Morita, T., Coen, P., Murthy, M., and Stern, D.L. (2013). Multi-channel acoustic recording and automated analysis of *Drosophila* courtship songs. BMC Biol. *11*, 1741-7007.

S2. Cachero, S., Ostrovsky, A.D., Yu, J.Y., Dickson, B.J., and Jefferis, G.S. (2010). Sexual dimorphism in the fly brain. Curr. Biol. *20*, 1589-1601.

S3. Jefferis, G.S., Potter, C.J., Chan, A.M., Marin, E.C., Rohlfing, T., Maurer, C.R., Jr., and Luo, L. (2007). Comprehensive maps of *Drosophila* higher olfactory centers: spatially segregated fruit and pheromone representation. Cell *128*, 1187-1203.

S4. Ostrovsky, A., Cachero, S., and Jefferis, G. (2013). Clonal analysis of olfaction in *Drosophila*: image registration. Cold Spring Harb Protoc. *2013*, 347-349.

S5. Zhou, L., Schnitzler, A., Agapite, J., Schwartz, L.M., Steller, H., and Nambu, J.R. (1997). Cooperative functions of the reaper and head involution defective genes in the programmed cell death of *Drosophila* central nervous system midline cells. Proc Natl Acad Sci U S A *94*, 5131-5136.

S6. McGuire, S.E., Le, P.T., Osborn, A.J., Matsumoto, K., and Davis, R.L. (2003). Spatiotemporal rescue of memory dysfunction in *Drosophila*. Science *302*, 1765-1768.

S7. Zhou, C., Pan, Y., Robinett, C.C., Meissner, G.W., and Baker, B.S. (2014). Central brain neurons expressing *doublesex* regulate female receptivity in *Drosophila*. Neuron *83*, 149-163.

S8. Pan, Y., Meissner, G.W., and Baker, B.S. (2012). Joint control of *Drosophila* male courtship behavior by motion cues and activation of male-specific P1 neurons. Proc Natl Acad Sci U S A *109*, 10065-10070.

S9. Rideout, E.J., Dornan, A.J., Neville, M.C., Eadie, S., and Goodwin, S.F. (2010). Control of sexual differentiation and behavior by the *doublesex* gene in *Drosophila melanogaster*. Nat Neurosci. *13*, 458-466.

S10. Rezaval, C., Nojima, T., Neville, M.C., Lin, A.C., and Goodwin, S.F. (2014). Sexually dimorphic octopaminergic neurons modulate female postmating behaviors in *Drosophila*. Curr. Biol. *24*, 725-730.

S11. Asahina, K., Watanabe, K., Duistermars, B.J., Hoopfer, E., Gonzalez, C.R., Eyjolfsdottir, E.A., Perona, P., and Anderson, D.J. (2014). Tachykinin-expressing neurons control male-specific aggressive arousal in *Drosophila*. Cell *156*, 221-235.

S12. Billeter, J.C., Atallah, J., Krupp, J.J., Millar, J.G., and Levine, J.D. (2009). Specialized cells tag sexual and species identity in *Drosophila melanogaster*. Nature *461*, 987-991.

S13. Pfeiffer, B.D., Ngo, T.T., Hibbard, K.L., Murphy, C., Jenett, A., Truman, J.W., and Rubin, G.M. (2010). Refinement of tools for targeted gene expression in *Drosophila*. Genetics *186*, 735-755.

S14. Stockinger, P., Kvitsiani, D., Rotkopf, S., Tirian, L., and Dickson, B.J. (2005). Neural

circuitry that governs Drosophila male courtship behavior. Cell *121*, 795-807.

S15. Yu, J.Y., Kanai, M.I., Demir, E., Jefferis, G.S., and Dickson, B.J. (2010). Cellular organization of the neural circuit that drives *Drosophila* courtship behavior. Curr. Biol. *20*, 1602-1614.

S16. von Philipsborn, A.C., Liu, T., Yu, J.Y., Masser, C., Bidaye, S.S., and Dickson, B.J. (2011). Neuronal control of Drosophila courtship song. Neuron *69*, 509-522.

S17. Villella, A., Gailey, D.A., Berwald, B., Ohshima, S., Barnes, P.T., and Hall, J.C. (1997). Extended reproductive roles of the fruitless gene in *Drosophila melanogaster* revealed by behavioral analysis of new fru mutants. Genetics *147*, 1107-1130.

S18. Fan, P., Manoli, D.S., Ahmed, O.M., Chen, Y., Agarwal, N., Kwong, S., Cai, A.G., Neitz, J., Renslo, A., Baker, B.S., et al. (2013). Genetic and neural mechanisms that inhibit *Drosophila* from mating with other species. Cell *154*, 89-102.

S19. Dweck, H.K., Ebrahim, S.A., Thoma, M., Mohamed, A.A., Keesey, I.W., Trona, F., Lavista-Llanos, S., Svatos, A., Sachse, S., Knaden, M., et al. (2015). Pheromones mediating copulation and attraction in *Drosophila*. Proc Natl Acad Sci U S A *112*, E2829-2835.

S20. Gorczyca, M.H., J.C. (1987). The Insectavox, an integrated device for recording and amplifying courtship songs of *Drosophila*. Dros. Inf. Serv *66*, 4.
