# Supplementary figures and images for: Activation of Latent Courtship Circuitry in the Brain of Drosophila Females Induces Male-like Behaviors
Source: Curr Biol. 2016 Sep 26;26(18):2508–15. doi: 10.1016/j.cub.2016.07.021 (PMC5049544; doi:10.1016/j.cub.2016.07.021)

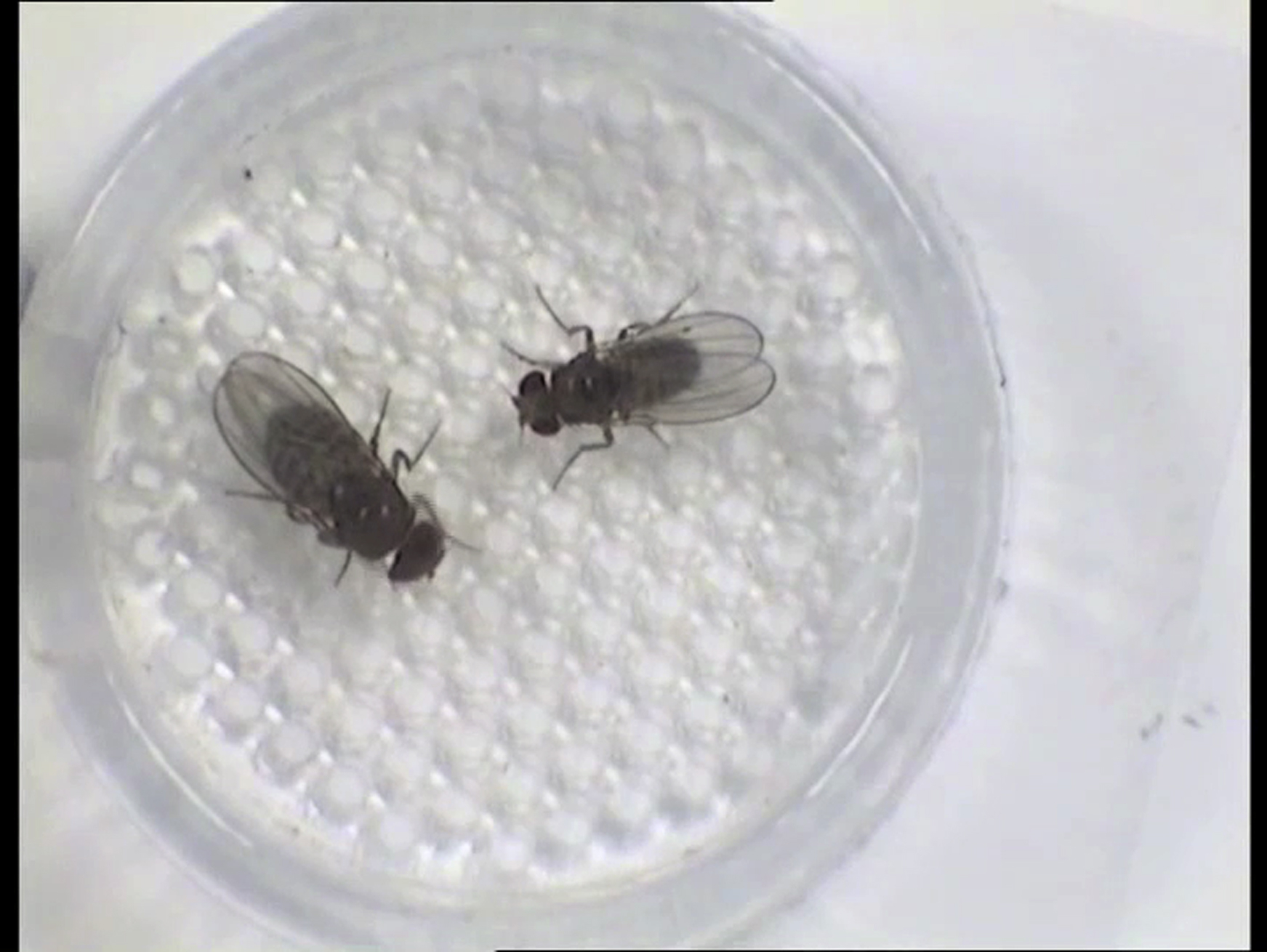

Supplement: Movie S1. dsxbrain>TrpA1 Females Are Normally Courted by a Wild-Type Male at 22°C, Related to Figure 1 — Movie showing a wild-type male displaying normal courtship behaviors towards a dsxbrain>TrpA1 female at the control temperature (22°C). [file mmc2.jpg]

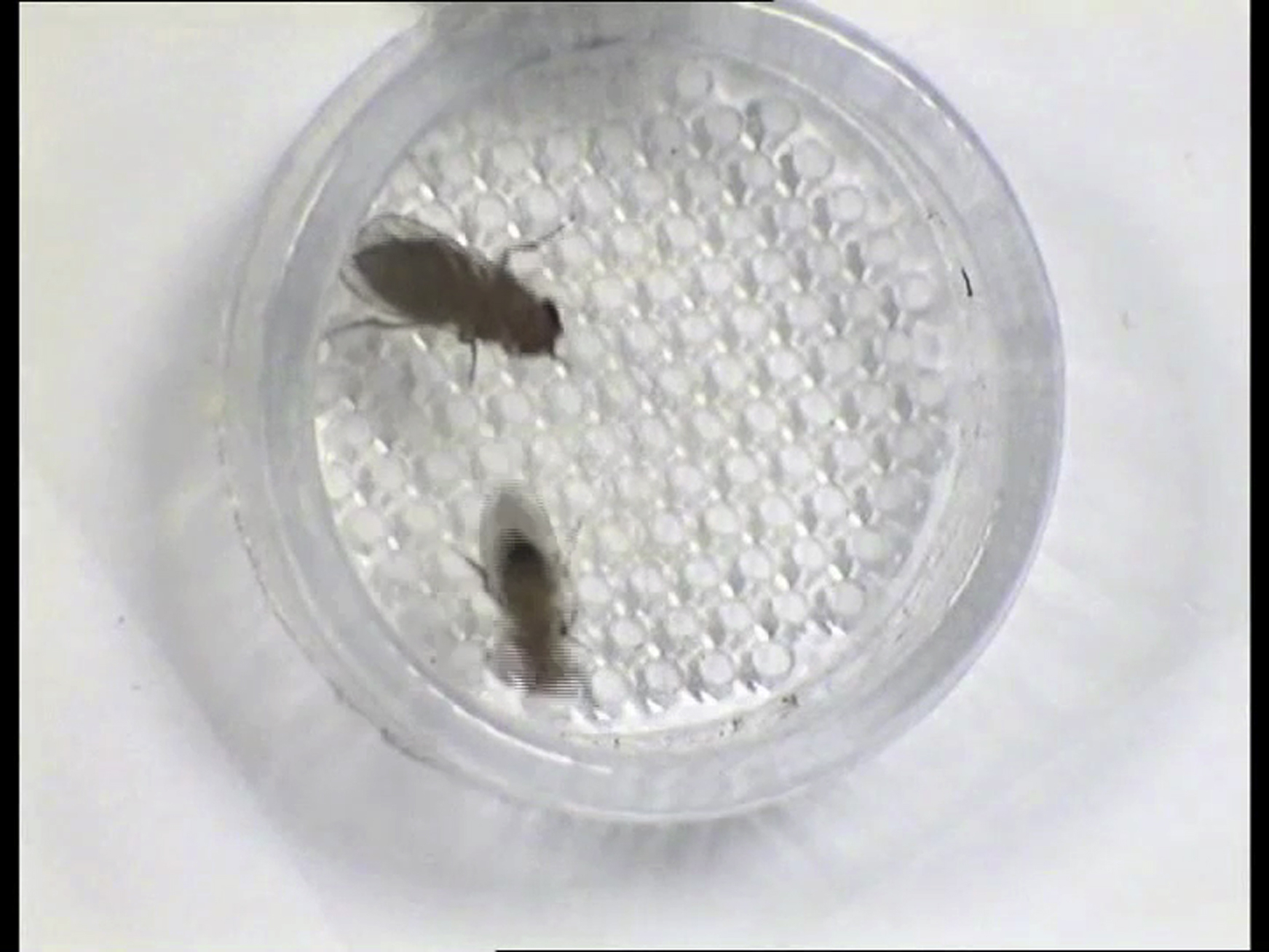

Supplement: Movie S2. Activation of dsx+ Neurons in Females Induces Male-like Courtship Behaviors toward a Wild-Type Male, Related to Figure 1 — Movie showing a dsxbrain>TrpA1 virgin female displaying male-typical courtship behaviors, such as following and wing extension, directed towards a wild-type male when thermally activated (at 33°C). [file mmc3.jpg]

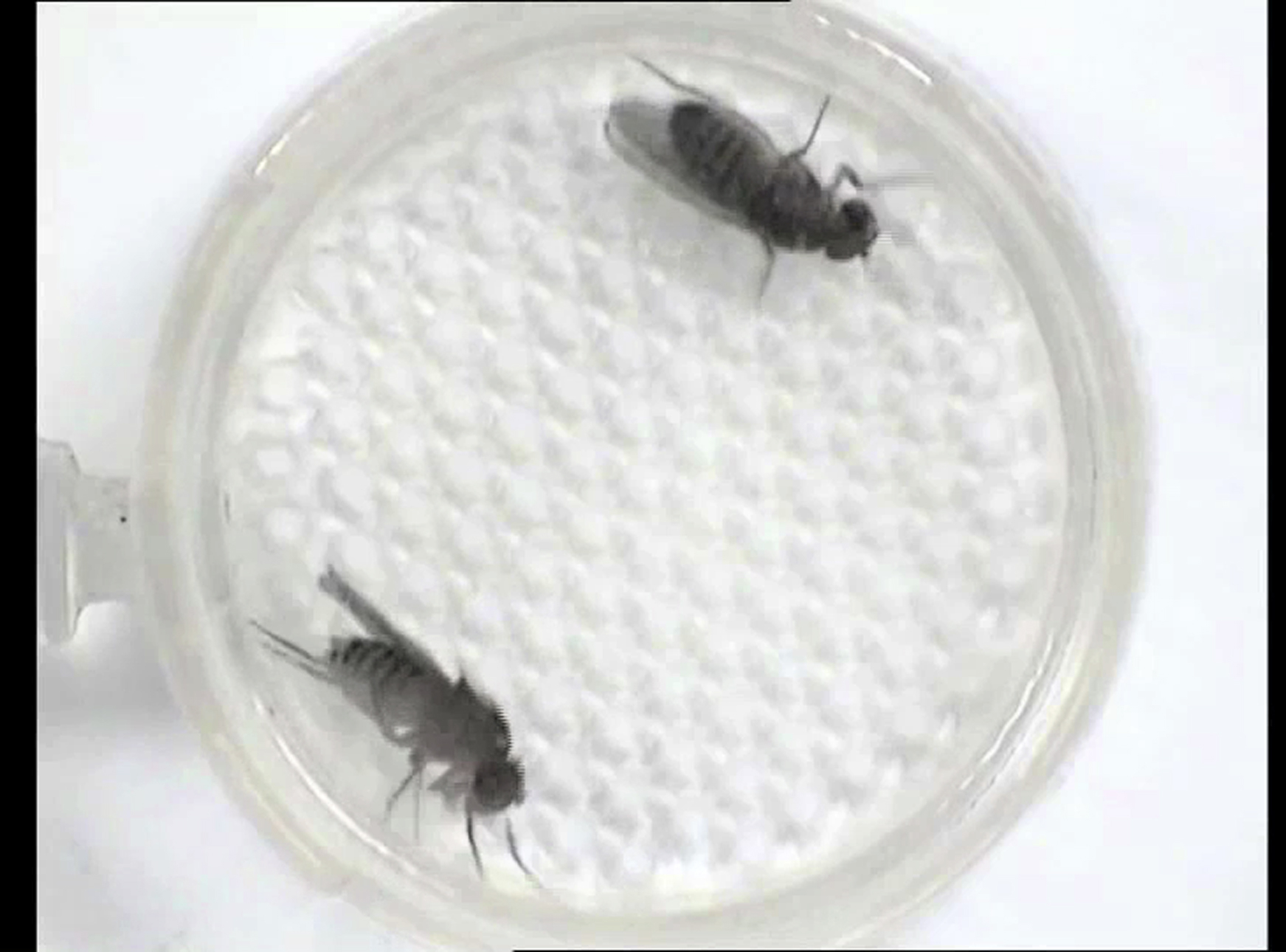

Supplement: Movie S3. Activation of dsx+ Neurons in Females Induces Male-like Courtship Behaviors toward a Wild-Type Female, Related to Figure 1 — Movie showing a dsxbrain>TrpA1 virgin female displaying male-typical courtship behaviors, such as following and wing extension, directed towards a wild-type female when thermally activated (at 33°C). [file mmc4.jpg]
