# Supplementary material for: Activation of Latent Courtship Circuitry in the Brain of Drosophila Females Induces Male-like Behaviors
Source: Curr Biol. 2016 Sep 26;26(18):2508–15. doi: 10.1016/j.cub.2016.07.021 (PMC5049544; doi:10.1016/j.cub.2016.07.021)
Supplement: Document S2. Article plus Supplemental Information [file mmc5.pdf]

Current Biology

Report

Activation of Latent Courtship Circuitry in the Brain of *Drosophila* Females Induces Male-like Behaviors

Highlights

- Activation of brain *dsx*-pC1 neurons promote male-like courtship in females
- Activated females court conspecific males and females and other *Drosophila* species
- Methyl pheromones trigger male courtship behaviors in activated females
- The female brain is equipped with latent circuitry underlying male-like behavior

Authors

Carolina Rezával, Siddharth Pattnaik, Hania J. Pavlou, ..., Luis A.D. D'Souza, Hany K.M. Dweck, Stephen F. Goodwin

Correspondence

carolina.rezaval@dpag.ox.ac.uk

In Brief

Rezával et al. found that activation of specific neurons in the brain induces female fruit flies to display male-like courtship behaviors and identified pheromones that induce such behaviors. Thus, this study shows that the female fly brain is equipped with latent courtship circuitry that is capable of inducing a male-specific behavioral program.

Rezával et al., 2016, Current Biology *26*, 2508–2515
September 26, 2016 © 2016 The Authors. Published by Elsevier Ltd.
http://dx.doi.org/10.1016/j.cub.2016.07.021

Current Biology
Report

Activation of Latent Courtship Circuitry in the Brain of *Drosophila* Females Induces Male-like Behaviors

Carolina Rezával,[1,3,*] Siddharth Pattnaik,[1] Hania J. Pavlou,[1] Tetsuya Nojima,[1] Birgit Brüggemeier,[1] Luis A.D. D'Souza,[1] Hany K.M. Dweck,[2] and Stephen F. Goodwin[1]
[1]Centre for Neural Circuits and Behaviour, University of Oxford, Tinsley Building, Mansfield Road, Oxford OX1 3SR, UK
[2]Department of Evolutionary Neuroethology, Max Planck Institute for Chemical Ecology, 07745 Jena, Germany
[3]Lead Contact
*Correspondence: carolina.rezaval@dpag.ox.ac.uk
http://dx.doi.org/10.1016/j.cub.2016.07.021

SUMMARY

Courtship in *Drosophila melanogaster* offers a powerful experimental paradigm for the study of innate sexually dimorphic behaviors [1, 2]. Fruit fly males exhibit an elaborate courtship display toward a potential mate [1, 2]. Females never actively court males, but their response to the male's display determines whether mating will actually occur. Sex-specific behaviors are hardwired into the nervous system via the actions of the sex determination genes *doublesex* (*dsx*) and *fruitless* (*fru*) [1]. Activation of male-specific *dsx/fru*$^+$ P1 neurons in the brain initiates the male's courtship display [3, 4], suggesting that neurons unique to males trigger this sex-specific behavior. In females, *dsx*$^+$ neurons play a pivotal role in sexual receptivity and post-mating behaviors [1, 2, 5–9]. Yet it is still unclear how *dsx*$^+$ neurons and dimorphisms in these circuits give rise to the different behaviors displayed by males and females. Here, we manipulated the function of *dsx*$^+$ neurons in the female brain to investigate higher-order neurons that drive female behaviors. Surprisingly, we found that activation of female *dsx*$^+$ neurons in the brain induces females to behave like males by promoting male-typical courtship behaviors. Activated females display courtship toward conspecific males or females, as well other *Drosophila* species. We uncovered specific *dsx*$^+$ neurons critical for driving male courtship and identified pheromones that trigger such behaviors in activated females. While male courtship behavior was thought to arise from male-specific central neurons, our study shows that the female brain is equipped with latent courtship circuitry capable of inducing this male-specific behavioral program.

RESULTS AND DISCUSSION

Brain-Restricted Activation of *dsx*$^+$ Neurons Induces Male Courtship Behaviors in Females

dsx is expressed in ~50 neurons in the brain and ~310 neurons in the ventral nerve cord (VNC) of females [5, 10–12]. To specifically assess the role of brain *dsx*$^+$ neurons in specifying female sexual behavior, we employed an intersectional approach, described in Figure 1A. We combined a *dsx*-specific Gal4 driver (*dsx*^{Gal4}) [5] and a brain-specifically expressed flippase recombinase (*Otd-FLP*) [13] with a Gal4/FLP-responsive reporter or effector. These *dsx*∩*Otd* intersected females (*dsx*^{brain}>*mGFP*) showed the typical *dsx* expression pattern in the brain but no expression in the VNC (Table S1; Figure 1B).

We assessed the behavioral effects of specifically activating brain *dsx*$^+$ neurons by expressing the heat-activated ion channel TrpA1, which induces depolarization when the temperature is increased above 25°C [14]. We paired a *dsx*^{brain}>*TrpA1* virgin female with a wild-type male at 22°C or 33°C (Figure 1C). At 22°C, *dsx*^{brain}>*TrpA1* virgin females behaved normally, with males actively courting them (Figure 1D; Movie S1). Surprisingly, when thermally activated (at 33°C), *dsx*^{brain}>*TrpA1* virgin females showed male-typical behaviors toward males (Figures 1E and S1; Movie S2). While wild-type *D. melanogaster* females never display male courtship behaviors, we found that *dsx*^{brain}>*TrpA1* females spend ~50% of their time courting males, performing the first steps of the behavioral ritual, such as following and tapping the target fly, as well as extending one or two wings (Figure S1). Activated females, however, did not engage in licking or abdominal bending associated with attempted copulation. Interestingly, most males targeted by the activated females showed greatly reduced levels of courtship and instead attempted to escape the female's advances (Figures 1E and S1; Movie S2). *dsx*^{brain}>*TrpA1* females were also attracted to wild-type females (Movie S3), showing no sex-specific bias in their behavioral response, as they spent a similar amount of time courting each sex in a preference assay (Figure 1F).

Activation of *dsx*$^+$ Neurons in the Female Brain Evokes Male-like Courtship Song

During courtship, *D. melanogaster* males vibrate one wing at a time producing a species-specific song that increases female receptivity [15, 16]. We thus tested whether *dsx*^{brain}>*TrpA1* females are capable of producing male-like courtship song. Audio recordings from activated females paired with wild-type males or females showed characteristic acoustic features of male-like courtship song (shown in Figure 2A), including sine and pulse

 © 2016 The Authors. Published by Elsevier Ltd.
This is an open access article under the CC BY license (http://creativecommons.org/licenses/by/4.0/).

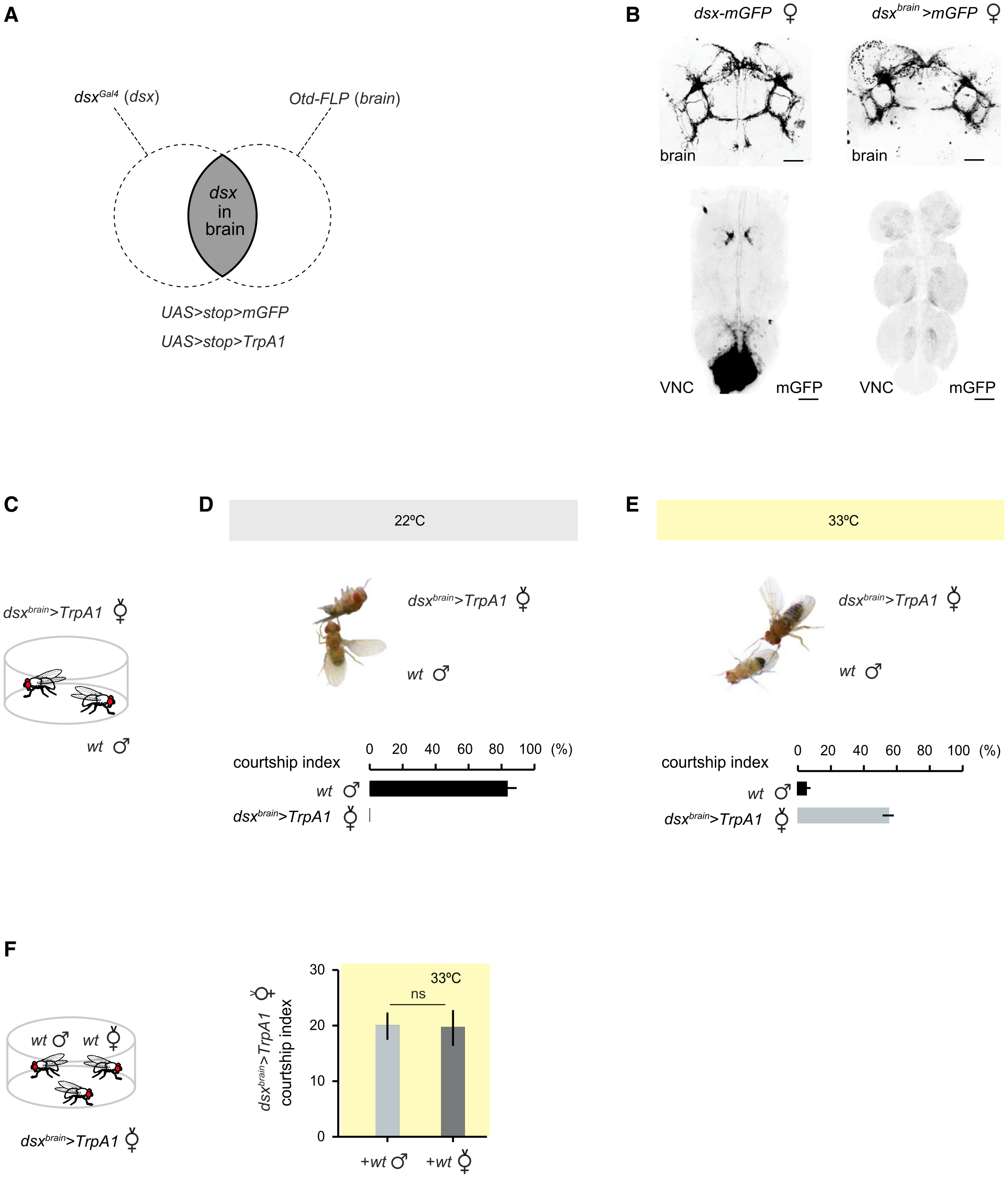

Figure 1. Activation of dsx^+ Neurons in the Female Brain Elicits Male-Typical Courtship Behaviors

(A) Intersectional strategy used to isolate dsx^+ neurons in the female brain. Brain-specific flippase (*Otd-FLP)* is used in combination with the *dsx*-specific driver dsx^{Gal4} and either *UAS>stop>mCD8::GFP* (mGFP) or *UAS>stop>TrpA1* (TrpA).

(B) Confocal images showing membrane-bound GFP expression in all dsx^+ neurons of the brain and VNC of females (*dsx-mGFP*; left) or intersected dsx^+ neurons in the brain (dsx^{brain}*>mGFP*; right). GFP staining is shown in black. Scale bars, 50 μm.

(C–E) Thermoactivation of dsx^+ neurons in the female brain.

(C) Courtship assay schematic: a *Otd-FLP/UAS>stop>TrpA1;*dsx^{Gal4}/+ virgin female (dsx^{brain}*>TrpA1*) was placed in a chamber with a wild-type (*wt*) male at the control (22°C) or activating temperature (33°C).

(D and E) Courtship assays for dsx^{brain}*>TrpA1* virgin females paired with wild-type males at 22°C (D) or 33°C (E). The courtship indices displayed by both males and females are reported. Courtship levels of wild-type males at 22°C are significantly higher than at 33°C and courtship levels of dsx^{brain}*>TrpA1* females at 33°C are significantly higher than at 22°C ($p < 0.0001$; Mann-Whitney test). n = 20.

(legend continued on next page)

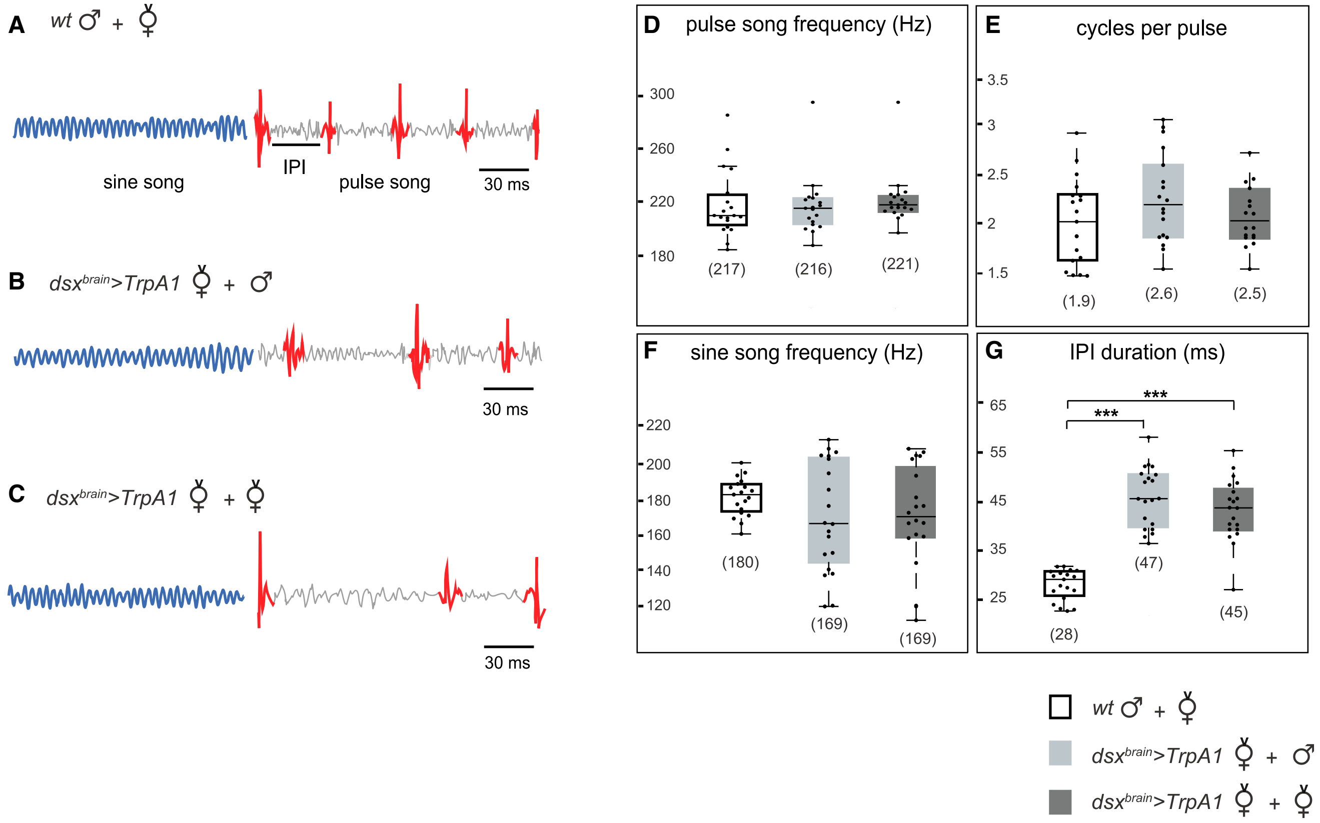

Figure 2. *dsx*$^{+}$ Neurons in the Female Brain Evoke Pulse and Sine Song

Courtship song was detected in recordings of 10 min. Sine song is shown in blue and pulses in red.

(A) Close up of courtship song trace produced by a wild-type male courting a wild-type virgin female at 33°C.

(B and C) Close up of courtship song traces produced by a *dsx*^{brain}*>TrpA1* female paired with a wild-type male (B) or female (C) at 33°C. Timescale indicated in milliseconds (ms).

(D–G) Boxplots of individual flies' pulse song frequency in Hz (D), number of cycles per pulse (E), sine song frequency in Hz (F), and mean IPI in ms (G). Median with interquartile range is indicated by boxes. Mean values are indicated below each box. n = 20 flies recorded. Kruskal-Wallis ANOVA was performed for all assays (***$p < 0.0001$). IPI, inter-pulse interval. See also Figure S2.

episodes (Figures 2B and 2C). How similar is activated female song to wild-type male song? The two have similar pulse and sine song frequency (Figures 2D and 2F) and number of cycles per pulse (Figure 2E). However, the inter-pulse interval (IPI), a species-specific parameter [16], was higher in activated female song than in wild-type male song (Figure 2G). We next quantified song events and examined bout structure in *dsx*^{brain}*>TrpA1* females. We found that activated females sing less song than males; both the number of sine bouts per minute and the duration of sine bouts were significantly reduced when compared to males (Figures S2D and S2E). Although the number of pulse bouts per minute did not differ between activated females and wild-type males (Figure S2F), the duration of pulse bouts was significantly shorter in females (Figure S2G). Thus, *dsx*^{brain}*>TrpA1* females display male-like pulse and sine song, albeit at much reduced levels compared to males. This is not surprising as male-specific *fru*$^{+}$ and *dsx*$^{+}$ neurons in the thorax contribute to song production [3, 17, 18]. In addition, a Dsx^{M}-dependent sexually dimorphic muscle is required for the production of robust sine song [17]. Photoactivation of *fru*$^{+}$ neurons in the thorax of headless females has previously been shown to elicit courtship song [19], leading to speculation that the motor program for song is present in females but lies dormant, because the neural commands in the brain required for song initiation are absent [3, 19]. In contrast, our data demonstrate that females possess brain neurons that, when activated, trigger song, as well as other male-like courtship behaviors.

dsx-pC1 Neurons Induce Male-Typical Courtship Behaviors in Females

dsx^{+} neurons in the female brain are distributed in discrete clusters: pC1, pC2, pC3 (also known as pCd [7]) and aDN [5, 10–12] (Figure 3A). As the female pC1 cluster is anatomically homologous to the male pC1 cluster [20], which includes the *fru*$^{+}$

(F) *dsx*^{brain}*>TrpA1* females do not display a sexual preference. Schematic of the courtship preference assay: a *dsx*^{brain}*>TrpA1* female was placed in a chamber with a male and a female at 33°C (left). Percentage of time a *dsx*^{brain}*>TrpA1* virgin female spent courting the male or female at 33°C (right). n = 20. A Mann-Whitney test was performed.

ns, not significant. Error bars, SEM. See also Figure S1 and Movies S1, S2, and S3.

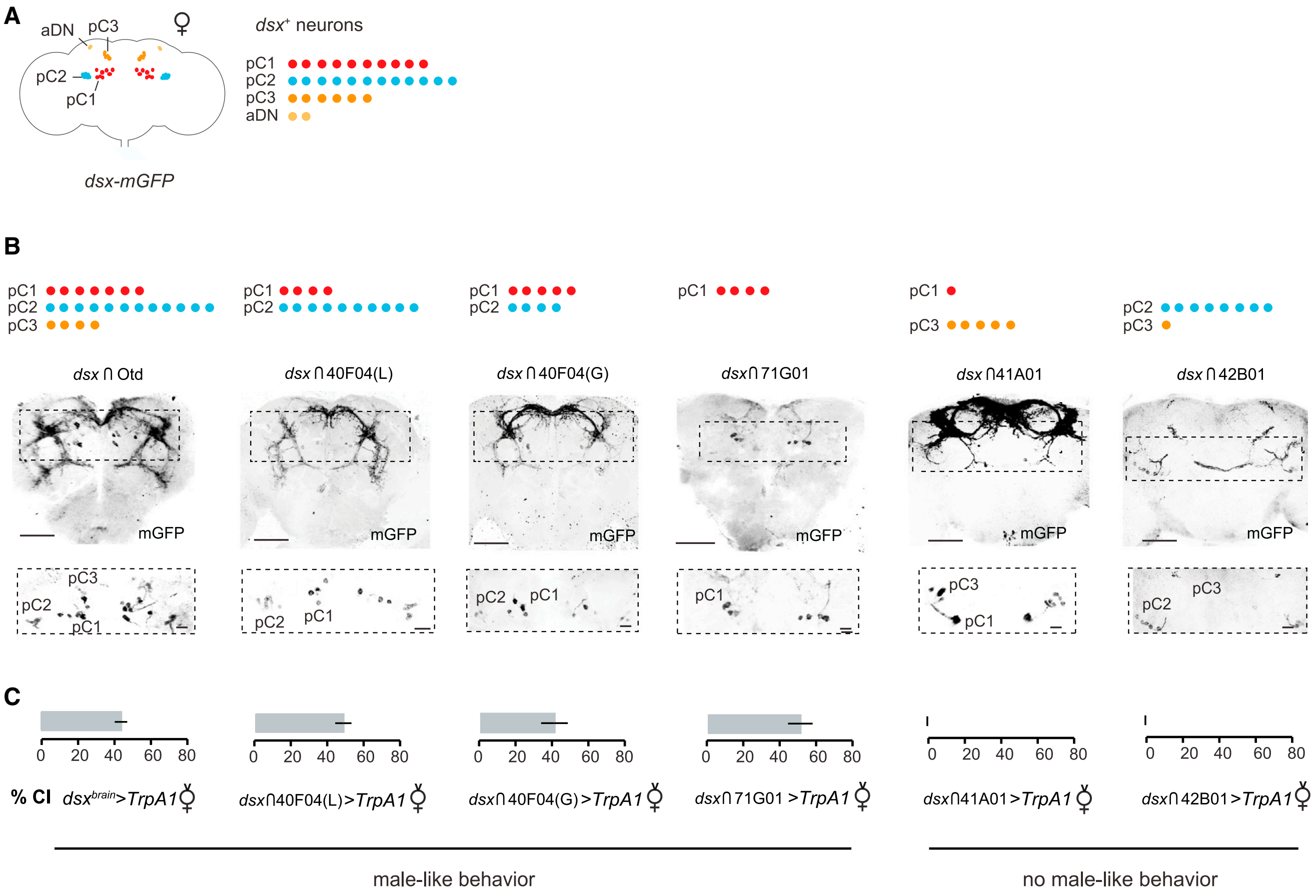

Figure 3. Mapping *dsx*$^{+}$ Brain Neuronal Clusters Critical for Inducing Male-like Behaviors in Females

(A) Schematic of *dsx*$^{+}$ neuronal clusters in the female brain labeled by GFP in control females (*dsx*^{Gal4}*/UAS-mGFP*).

(B) Genetic subdivision of *dsx*$^{+}$ neurons in the female brain combining different genetic tools (see Table S1) with *UAS>stop>mGFP*. mGFP expression is shown in black. The number of *dsx*$^{+}$ neurons found in each brain cluster per hemisegment (shown in Table S1) is represented with dots. Scale bars, 50 μm. Higher magnification of the dorsal brain is shown in the bottom panel. Scale bars, 12.5 μm.

(C) Behavioral effects of thermoactivating subsets of *dsx*$^{+}$ neurons in the female brain. The graphs depict the courtship index (CI) displayed by intersected females in the presence of a wild-type male at 33°C. n = 12–15. Error bars, SD. No significant differences were observed between different intersected females displaying male-like courtship ($p > 0.05$; Mann-Whitney test).

See also Figure S3.

courtship promoting P1 neurons [3, 4, 21, 22], we reasoned that this cluster may be responsible for the activation of male-typical courtship behaviors in *dsx*^{brain}*>TrpA1* females. To test this hypothesis, we exploited intersectional methods [5, 7, 8, 20, 23] to target distinct *dsx*$^{+}$ neuronal clusters in the female brain (Figures 3A and 3B; Table S1). We first visualized intersected *dsx*$^{+}$ neurons in the female and male nervous system (Figure 3B; Table S1). We next used these restricted lines to express TrpA1 to test whether activating distinct *dsx*$^{+}$ clusters drives male-like courtship behaviors in females (Figure 3C). We found that females only displayed male-like courtship behaviors toward males in strains that intersected four or more pC1 neurons per hemisphere. In contrast, females in which the majority of pC2 neurons or pC3 neurons are artificially activated did not show male-like behaviors. The clearest role for pC1 neurons in inducing male-like behaviors was seen in *dsx∩71G01* females, where only four pC1 neurons are activated; these females showed high levels of courtship when paired with a wild-type male (~50%; Figure 3C), displaying following, tapping, and wing extension behaviors (Figure S3A).

Activating the homologous neurons in *dsx∩71G01>TrpA1* males also triggered courtship behaviors (Figures S3B and S3C), consistent with previous findings [6, 20]. Unlike the corresponding females, these males display courtship behaviors in the presence or absence of a male or female, and at lower activating temperatures (30 versus 32°C; Figures S3J and S3K). Note that males have more pC1 neurons labeled by *dsx∩71G01* than females (~17 versus approximately four neurons per brain hemisphere, respectively) (Table S1; Figures S3F–S3I), consistent with previous findings [7]. pC1 neurons in *dsx∩71G01* males include both *dsx*$^{+}$/*fru*$^{-}$ neurons and *fru*$^{+}$/*dsx*$^{+}$ P1 neurons (data not shown), while pC1 neurons in females are all *dsx*$^{+}$/*fru*$^{-}$ [7, 9, 10] and are thus distinct from the *fru/dsx*$^{+}$ P1 subgroup in males. Thus, artificial activation of sexually dimorphic *dsx*-pC1 neurons promotes male courtship behaviors in both sexes.

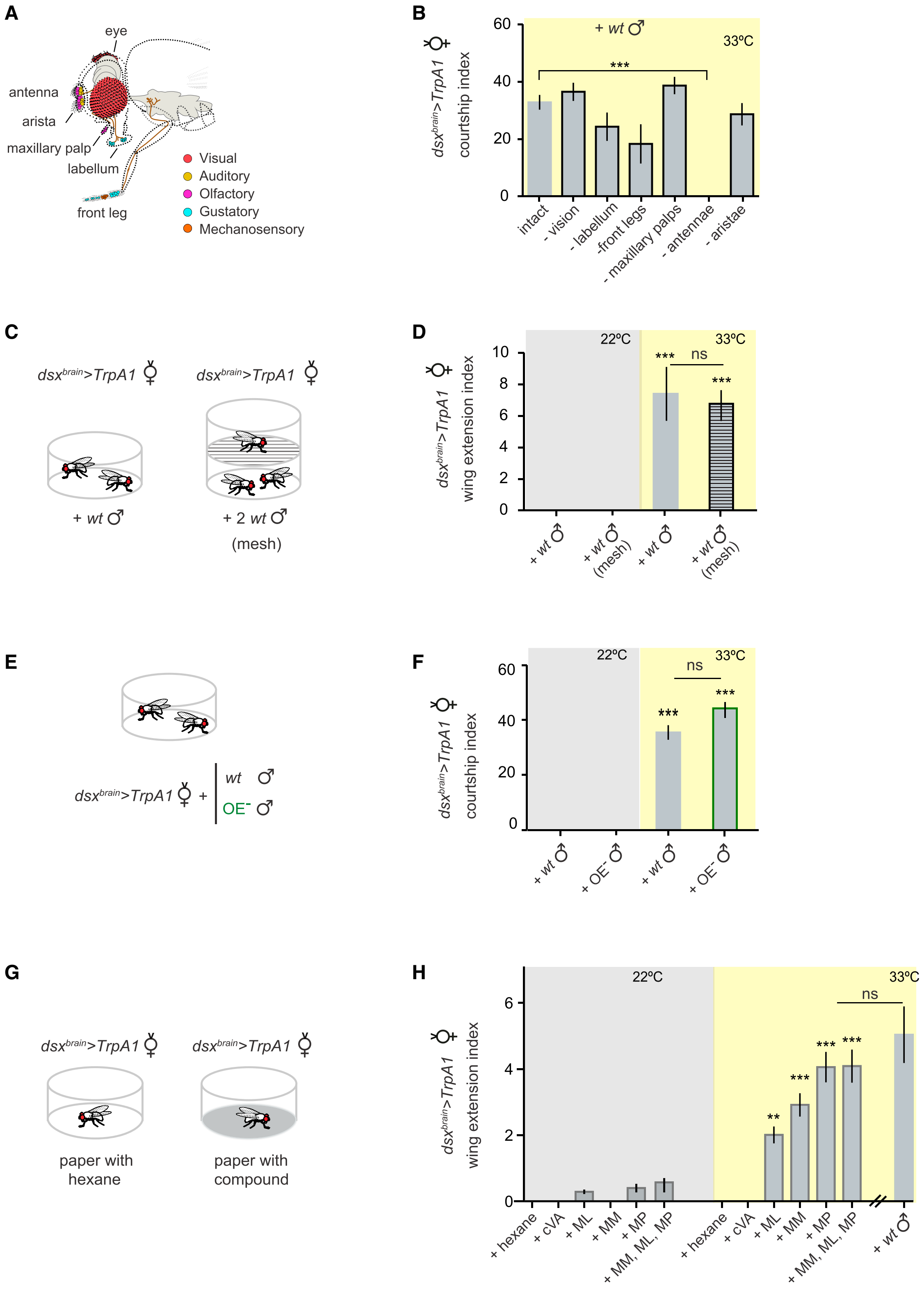

Figure 4. Identification of Pheromones Required for Inducing Male Courtship Behaviors in Females

(A) Schematic showing appendages and associated sensory modalities involved in courtship behaviors (modified from [25]).

(B) Courtship index displayed by *dsx*^{brain}>*TrpA1* females with intact or severed sensory modalities when paired with a wild-type male at 33°C. n = 20–30.

(legend continued on next page)

Volatile Compounds Induce Male Courtship Behaviors in Females

In contrast to dsx^{brain}>*TrpA1* males, dsx^{brain}>*TrpA1* females do not show courtship behaviors at 33°C in isolation (data not shown), suggesting that activated females require additional sensory cues provided by another fly to trigger courtship behaviors. To identify such sensory cues, we disrupted individual sensory modalities known to stimulate courtship behavior in males, in dsx^{brain}>*TrpA1* females [2, 24] (Figure 4A). To block vision in general, we paired dsx^{brain}>*TrpA1* females with headless males in the dark. To block gustation, we removed the females' labella or front legs. To block audition, we removed the females' aristae. In all of these cases, dsx^{brain}>*TrpA1* females still showed male-like courtship behaviors (Figure 4B). In contrast, when we blocked olfaction by removing the antennae, the dsx^{brain}>*TrpA1* females did not court, suggesting that olfactory cues contribute to the induction of male-like behaviors (Figure 4B). Moreover, when we placed dsx^{brain}>*TrpA1* females in close proximity to a pair of males, without allowing physical contact, the activated females showed courtship-specific wing extension (Figures 4C and 4D), further indicating that male-like courtship in dsx^{brain}>*TrpA1* females can be induced by volatile olfactory cues. Interestingly, removing the maxillary palps did not block courtship behavior (Figure 4B), suggesting that the relevant olfactory cues are detected by the antennae, not the maxillary palps.

Methyl Laurate, Methyl Myristate, and Methyl Palmitate Are Stimulatory Pheromones for Male-like Behaviors in Females

The finding that dsx^{brain}>*TrpA1* females court both males and females (Figure 1F) suggested male-like behaviors might be induced by pheromones common to both sexes. Oenocyte cells (OEs) are the main sites of pheromone production in *Drosophila*, as ablation of these cells eliminates cuticular hydrocarbons, which act as pheromones [24, 26]. Surprisingly, males with and without OE cells elicited similar courtship levels in activated females (Figures 4E and 4F), suggesting that the pheromones that stimulate courtship in activated females are not produced by oenocytes. The only known volatile pheromone found in OE-less males is 11-cis-Vaccenyl acetate (cVA), as it is produced in the male ejaculatory bulb [27, 28]. However, cVA is missing in virgin females [29]; thus, it cannot explain why activated dsx^{brain}>*TrpA1* females are stimulated to court virgin females. Indeed, cVA did not induce wing extension in solitary dsx^{brain}>*TrpA1* females at 33°C (Figures 4G and 4H).

It was recently shown that methyl laurate (ML), methyl myristate (MM), and methyl palmitate (MP) are cuticular odorants that elicit short-range attraction behavior in both sexes and are present approximately equally in males and females [30]. Interestingly, ML, MM, and MP are found at normal levels in oenocyte-less flies as well as in other *Drosophila* species [30]. We tested whether exposing solitary dsx^{brain}>*TrpA1* females to candidate odors promoted wing extension at 33°C. Whereas hexane, the solvent used to dilute the compounds, did not elicit wing extension, ML, MM, or MP all triggered this behavior (Figures 4G and 4H). In particular, MP triggered the highest levels of wing extension in activated females in our assay, which were similar to the levels elicited by the three compounds together (Figure 4H). These cuticular compounds appear to act at short distances in dsx^{brain}>*TrpA1* females, as these females are generally in close contact with their courtship targets, and show decreased courtship levels in bigger chambers (<29% versus >43% courtship index, $n = 25$, $p < 0.05$).

We next asked whether dsx^{brain}>*TrpA1* females could be stimulated by other *Drosophila* species [31, 32], which are not normally courted by *D. melanogaster* males [33]. We found that ~60% of activated dsx^{brain}>*TrpA1* females courted flies of related species within the *melanogaster subgroup* (*D. simulans* and *D. yakuba*), and more distantly related species within the subgenus *Sophophora* (*D. wilistoni*) (Figures S4A and S4B). Only ~20%–30% of activated females showed courtship toward *D. virilis* flies, which are distantly related to *D. melanogaster*, having diverged about 60 million years ago [32]. While *D. melanogaster*, *D. simulans*, *and D. yakuba* produce either MP and/or ML and MM [30], we found that none of these methyl pheromones are present in *D. virilis* flies (Figure S4C). Interestingly, coating *D. virilis* males with MP increased both the number of dsx^{brain}>*TrpA1* females displaying male courtship behaviors (~56%) and the courtship levels when compared to hexane-treated *D. virilis* flies (Figure S4D). Our data support a role for MP as an important stimulatory olfactory cue for male courtship behaviors in dsx^{brain}>*TrpA1* females.

Sexually Dimorphic Usage of Common Circuitry

If females have neurons capable of inducing courtship, why do they not normally display these male behaviors? Two observations may be instructive: first, females require more stimulation—both sensory and thermogenetic—to show male courtship behavior. *dsx*∩*71G01*>*dTrpA1* females require higher temperatures than males, in addition to olfactory cues, to show activation-induced courtship (Figures S3A–S3C, S3J, and S3K). Second, stimulation of pC1 neurons in

(C and D) Volatile olfactory cues trigger male-like behaviors in dsx^{brain}>*TrpA1* females. (C) Schematic of assay in which a dsx^{brain}>*TrpA1* female is paired either with a male in the same courtship chamber or separated from two males by mesh. (D) Courtship index displayed by dsx^{brain}>*TrpA1* virgin females in contact or without contact (+ mesh) with male targets at 22°C (gray box) or 33°C (yellow box). n = 20.

(E–H) Identification of pheromones promoting male-like behaviors in dsx^{brain}>*TrpA1* females.

(E) A dsx^{brain}>*TrpA1* female was placed in a courtship chamber with either a male (*wt*) or an oenocyte-less male (OE^{-}).

(F) Courtship index displayed by dsx^{brain}>*TrpA1* females paired with target flies at 22°C (gray box) or 33°C (yellow box).

(G) Schematic of assay in which a dsx^{brain}>*TrpA1* female is placed in a courtship chamber containing a filter paper soaked with either hexane (solvent), 11-cis-Vaccenyl acetate (cVA), methyl laurate (ML), methyl myristate (MM), methyl palmitate (MP) or a ML, MM, and MP mix.

(H) Wing extension index displayed by a solitary dsx^{brain}>*TrpA1* virgin female in the presence of each compound at 22°C (gray box) or 33°C (yellow box). (n = 20). Statistical comparisons of the indicated genotypes at 33°C were made against genotypes at 22°C unless otherwise indicated. A Kruskal-Wallis ANOVA test was performed in (B), (D), (F), and (H) (**$p < 0.001$; ***$p < 0.0001$). Error bars, SEM; ns, not significant. Courtship behaviors were measured over 3 min. See also Figure S4.

dsx∩*71G01>dTrpA1* females induces different behaviors depending on the level of stimulation, which presumably correlates with heat-induced changes in the neural activity of pC1 neurons [14, 34, 35]). We found that raising the temperature from 22°C to 27°C increases female receptivity, seen in their latency to copulation (670 ± 50 s versus 1,452 ± 175 s, n = 20, $p < 0.05$) and their ability to elicit higher levels of male courtship (~90% versus ~66% courtship index, n = 20, $p < 0.001$). This is consistent with a previous study showing pC1 neurons are responsive to courtship-relevant stimuli, such as cVA and courtship song, and promote receptivity when activated at low temperatures [7]. We further found that raising the temperature from 27°C to 33°C causes females to transition from female-typical behaviors to male-typical behaviors (Figure S3L).

The reason males court, while females do not, may be due to an intrinsic difference in this key node in the courtship circuit. This could be a consequence of (1) differences in the number of pC1 neurons (e.g., fewer neurons in females are not sufficient to trigger male courtship behaviors), (2) differences in the intrinsic properties of pC1 neurons (e.g., they are less excitable in females), and/or (3) differences in the connectivity of pC1 neurons (e.g., they receive more inhibition or less excitation in females). Discrete pC1 neurons may function as a switch for courtship versus aggression in males, with fru^{+}/dsx^{+} neurons mediating courtship and fru^{-}/dsx^{+} mediating aggression [22]. Thus, the pC1 node in both sexes appears to integrate sensory cues to regulate socio-sexual behavior (courtship and aggression in males [3, 4, 21, 22, 36], receptivity in females [7]), but when it is strongly activated outside the physiological range in females, it can activate downstream circuitry (e.g., fru^{+} thoracic neurons [3, 19]) for courtship that normally lies dormant.

We have identified MP and related cuticular pheromones as stimulators of male-like behaviors in females. Interestingly, MP also triggers male courtship behaviors in solitary activated males (Figures S3D and S3E). ML, MM, and MP induce attraction behavior through Or88a olfactory neurons in the antenna, which can facilitate mate finding [30]. In addition, ML stimulates male copulation behavior via Or47b olfactory neurons [30, 37]. Thus, Or88a and Or47b-expressing neurons are strong candidates for mediating male courtship behaviors in dsx^{brain}>*TrpA1* females.

How are pC1 activation and MP stimulation integrated? This cuticular pheromone might modulate the activity of pC1 neurons or, alternatively, pheromone-responsive circuits and pC1 neurons might converge on downstream neurons that mediate courtship behaviors in females and males. It should be noted that, since pC1 neurons are probably maximally activated at 33°C in our experiments (Figures S3J and S3K), MP is unlikely to further activate these neurons. This suggests that pC1 activation and MP stimulation may be integrated in downstream neurons.

It was previously proposed that male courtship behavior, such as song, may result from the activation of brain neurons unique to males [3, 19]. Here, we show that the female brain is capable of inducing male-like behaviors. Notably, a male-sexual behavior effector circuit has been shown to be present in the brain of female mice, which is normally repressed [38]. Hence, the existence of functional neuronal circuitry underlying male-specific behaviors in the normal female brain seems to be a conserved feature of courtship circuit organization [39].

SUPPLEMENTAL INFORMATION

Supplemental Information includes Supplemental Experimental Procedures, four figures, one table, and three movies and can be found with this article online at http://dx.doi.org/10.1016/j.cub.2016.07.021.

AUTHOR CONTRIBUTIONS

C.R., S.P., B.B., and S.F.G. designed the experiments and contributed to data analysis, interpretation, and discussion. C.R., S.P., H.J.P., T.N., B.B., L.A.D.D., and H.K.M.D. conducted the experiments. The manuscript was written by C.R. and corrected by S.F.G. All authors contributed to the revision of the final manuscript.

ACKNOWLEDGMENTS

We thank Megan Neville, Andrew Lin, Luis Suarez, Annika Rings, Sofia Lavista-Llanos, and Ana Depetris Chauvin for helpful discussions and critical reading of the manuscript. We are also grateful to Joel Levine, Barry Dickson, David Anderson, Mike Ritchie, and Gerry Rubin for sharing important reagents with us. We thank Gregory Jefferis for advice on image registration. This work was supported by grants from the BBSRC (BB/N000803/1) to C.R., and S.F.G., and a Wellcome Trust Investigator Award to S.F.G (WT106189MA).

Received: March 28, 2016
Revised: June 13, 2016
Accepted: July 12, 2016
Published: August 25, 2016

REFERENCES

1. Pavlou, H.J., and Goodwin, S.F. (2013). Courtship behavior in *Drosophila melanogaster*: towards a "courtship connectome". Curr. Opin. Neurobiol. *23*, 76–83.
2. Auer, T.O., and Benton, R. (2016). Sexual circuitry in *Drosophila*. Curr. Opin. Neurobiol. *38*, 18–26.
3. von Philipsborn, A.C., Liu, T., Yu, J.Y., Masser, C., Bidaye, S.S., and Dickson, B.J. (2011). Neuronal control of *Drosophila* courtship song. Neuron *69*, 509–522.
4. Kohatsu, S., Koganezawa, M., and Yamamoto, D. (2011). Female contact activates male-specific interneurons that trigger stereotypic courtship behavior in *Drosophila*. Neuron *69*, 498–508.
5. Rideout, E.J., Dornan, A.J., Neville, M.C., Eadie, S., and Goodwin, S.F. (2010). Control of sexual differentiation and behavior by the *doublesex* gene in *Drosophila melanogaster*. Nat. Neurosci. *13*, 458–466.
6. Kimura, K., Sato, C., Koganezawa, M., and Yamamoto, D. (2015). *Drosophila* ovipositor extension in mating behavior and egg deposition involves distinct sets of brain interneurons. PLoS ONE *10*, e0126445.
7. Zhou, C., Pan, Y., Robinett, C.C., Meissner, G.W., and Baker, B.S. (2014). Central brain neurons expressing *doublesex* regulate female receptivity in *Drosophila*. Neuron *83*, 149–163.
8. Rezával, C., Nojima, T., Neville, M.C., Lin, A.C., and Goodwin, S.F. (2014). Sexually dimorphic octopaminergic neurons modulate female postmating behaviors in *Drosophila*. Curr. Biol. *24*, 725–730.
9. Rezával, C., Pavlou, H.J., Dornan, A.J., Chan, Y.B., Kravitz, E.A., and Goodwin, S.F. (2012). Neural circuitry underlying *Drosophila* female postmating behavioral responses. Curr. Biol. *22*, 1155–1165.
10. Sanders, L.E., and Arbeitman, M.N. (2008). Doublesex establishes sexual dimorphism in the *Drosophila* central nervous system in an isoform-dependent manner by directing cell number. Dev. Biol. *320*, 378–390.

11. Lee, G., Hall, J.C., and Park, J.H. (2002). *Doublesex* gene expression in the central nervous system of *Drosophila melanogaster*. J. Neurogenet. *16*, 229–248.

12. Robinett, C.C., Vaughan, A.G., Knapp, J.M., and Baker, B.S. (2010). Sex and the single cell. II. There is a time and place for sex. PLoS Biol. *8*, e1000365.

13. Asahina, K., Watanabe, K., Duistermars, B.J., Hoopfer, E., González, C.R., Eyjólfsdóttir, E.A., Perona, P., and Anderson, D.J. (2014). Tachykinin-expressing neurons control male-specific aggressive arousal in *Drosophila*. Cell *156*, 221–235.

14. Hamada, F.N., Rosenzweig, M., Kang, K., Pulver, S.R., Ghezzi, A., Jegla, T.J., and Garrity, P.A. (2008). An internal thermal sensor controlling temperature preference in *Drosophila*. Nature *454*, 217–220.

15. Von Schilcher, F. (1976). The behavior of cacophony, a courtship song mutant in *Drosophila melanogaster*. Behav. Biol. *17*, 187–196.

16. Bennet-Clark, H.C.E.A.W. (1969). Pulse interval as a critical parameter in the courtship song of *Drosophila melanogaster*. Anim. Behav. *17*, 755–759.

17. Shirangi, T.R., Stern, D.L., and Truman, J.W. (2013). Motor control of *Drosophila* courtship song. Cell Rep. *5*, 678–686.

18. Shirangi, T.R., Wong, A.M., Truman, J.W., and Stern, D.L. (2016). Doublesex regulates the connectivity of a neural circuit controlling *Drosophila* male courtship song. Dev. Cell *37*, 533–544.

19. Clyne, J.D., and Miesenböck, G. (2008). Sex-specific control and tuning of the pattern generator for courtship song in *Drosophila*. Cell *133*, 354–363.

20. Pan, Y., Meissner, G.W., and Baker, B.S. (2012). Joint control of *Drosophila* male courtship behavior by motion cues and activation of male-specific P1 neurons. Proc. Natl. Acad. Sci. USA *109*, 10065–10070.

21. Kimura, K., Hachiya, T., Koganezawa, M., Tazawa, T., and Yamamoto, D. (2008). *Fruitless* and *doublesex* coordinate to generate male-specific neurons that can initiate courtship. Neuron *59*, 759–769.

22. Koganezawa, M., Kimura, K., and Yamamoto, D. (2016). The neural circuitry that functions as a witch for courtship versus aggression in *Drosophila* males. Curr. Biol. *26*, 1395–1403.

23. Venken, K.J., Simpson, J.H., and Bellen, H.J. (2011). Genetic manipulation of genes and cells in the nervous system of the fruit fly. Neuron *72*, 202–230.

24. Kohl, J., Huoviala, P., and Jefferis, G.S. (2015). Pheromone processing in *Drosophila*. Curr. Opin. Neurobiol. *34*, 149–157.

25. Hartenstein, V. (1993). Atlas of *Drosophila* Development (Cold Spring Harbor Laboratory Press).

26. Billeter, J.C., Atallah, J., Krupp, J.J., Millar, J.G., and Levine, J.D. (2009). Specialized cells tag sexual and species identity in *Drosophila melanogaster*. Nature *461*, 987–991.

27. Butterworth, F.M. (1969). Lipids of *Drosophila*: a newly detected lipid in the male. Science *163*, 1356–1357.

28. Brieger, G., and Butterworth, F.M. (1970). *Drosophila melanogaster*: identity of male lipid in reproductive system. Science *167*, 1262.

29. Ejima, A., Smith, B.P., Lucas, C., van der Goes van Naters, W., Miller, C.J., Carlson, J.R., Levine, J.D., and Griffith, L.C. (2007). Generalization of courtship learning in *Drosophila* is mediated by cis-vaccenyl acetate. Curr. Biol. *17*, 599–605.

30. Dweck, H.K., Ebrahim, S.A., Thoma, M., Mohamed, A.A., Keesey, I.W., Trona, F., Lavista-Llanos, S., Svatoš, A., Sachse, S., Knaden, M., and Hansson, B.S. (2015). Pheromones mediating copulation and attraction in *Drosophila*. Proc. Natl. Acad. Sci. USA *112*, E2829–E2835.

31. Ko, W.Y., David, R.M., and Akashi, H. (2003). Molecular phylogeny of the *Drosophila* melanogaster species subgroup. J. Mol. Evol. *57*, 562–573.

32. Russo, C.A., Takezaki, N., and Nei, M. (1995). Molecular phylogeny and divergence times of drosophilid species. Mol. Biol. Evol. *12*, 391–404.

33. Fan, P., Manoli, D.S., Ahmed, O.M., Chen, Y., Agarwal, N., Kwong, S., Cai, A.G., Neitz, J., Renslo, A., Baker, B.S., and Shah, N.M. (2013). Genetic and neural mechanisms that inhibit *Drosophila* from mating with other species. Cell *154*, 89–102.

34. Pulver, S.R., Pashkovski, S.L., Hornstein, N.J., Garrity, P.A., and Griffith, L.C. (2009). Temporal dynamics of neuronal activation by Channelrhodopsin-2 and TRPA1 determine behavioral output in *Drosophila* larvae. J. Neurophysiol. *101*, 3075–3088.

35. Berni, J., Muldal, A.M., and Pulver, S.R. (2010). Using neurogenetics and the warmth-gated ion channel TRPA1 to study the neural basis of behavior in *Drosophila*. J. Undergrad. Neurosci. Educ. *9*, A5–A14.

36. Hoopfer, E.D., Jung, Y., Inagaki, H.K., Rubin, G.M., and Anderson, D.J. (2015). P1 interneurons promote a persistent internal state that enhances inter-male aggression in *Drosophila*. eLife *4*, e11346.

37. Lin, H.H., Cao, D.S., Sethi, S., Zeng, Z., Chin, J.S., Chakraborty, T.S., Shepherd, A.K., Nguyen, C.A., Yew, J.Y., Su, C.Y., and Wang, J.W. (2016). Hormonal modulation of pheromone detection enhances male courtship success. Neuron *90*, 1272–1285.

38. Kimchi, T., Xu, J., and Dulac, C. (2007). A functional circuit underlying male sexual behaviour in the female mouse brain. Nature *448*, 1009–1014.

39. Dulac, C., and Kimchi, T. (2007). Neural mechanisms underlying sex-specific behaviors in vertebrates. Curr. Opin. Neurobiol. *17*, 675–683.

Current Biology, Volume *26*

Supplemental Information

Activation of Latent Courtship Circuitry

in the Brain of *Drosophila* Females

Induces Male-like Behaviors

Carolina Rezával, Siddharth Pattnaik, Hania J. Pavlou, Tetsuya Nojima, Birgit Brüggemeier, Luis A.D. D'Souza, Hany K.M. Dweck, and Stephen F. Goodwin

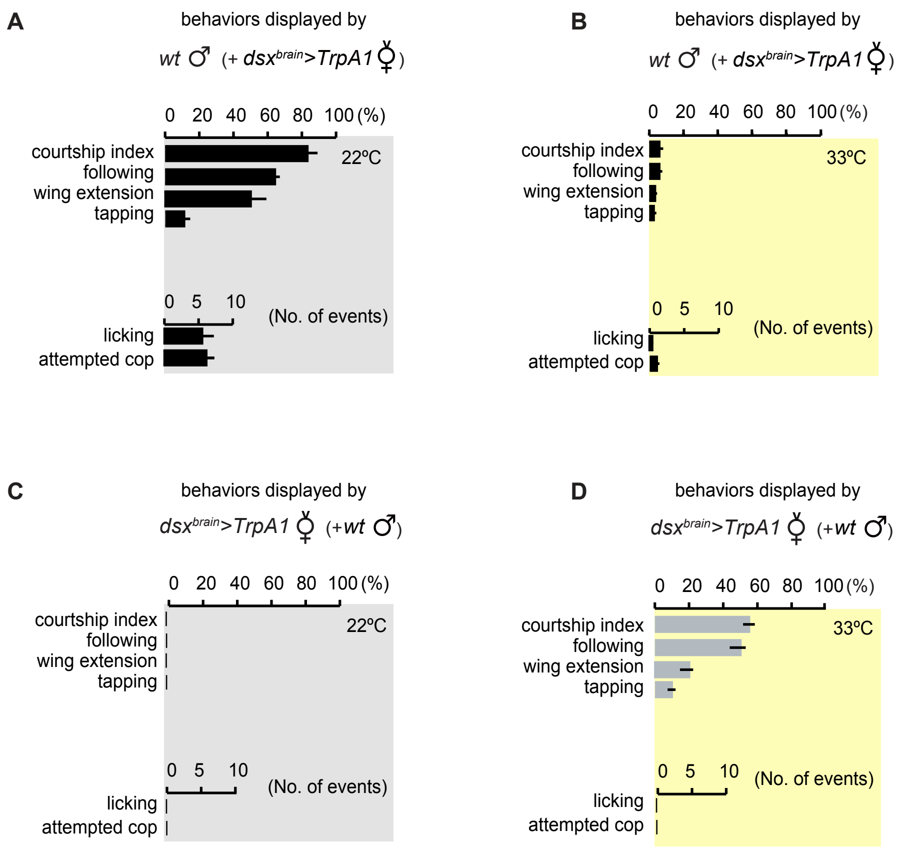

Figure S1, related to Figure 1. Behavioral characterization of *dsx*^{brain}*>TrpA1* females and wild-type males. Courtship index displayed by wild-type (*wt*) males paired with *Otd-FLP/UAS>stop>TrpA1;dsx*^{Gal4}/+ virgin females (indicated as *dsx*^{brain}*>TrpA1*) at 22°C (grey panel; **A**) or 33°C (yellow panel; **B**). Courtship index displayed by *dsx*^{brain}*>TrpA1* virgin females paired with *wt* males at 22°C (grey panel; **C**) or 33°C (yellow panel; **D**). Flies were recorded at high magnification to monitor individual courtship steps. For following, wing extension and tapping the total time engaged in these steps was recorded; for licking and attempted copulation (attempted cop), it was the total number of events. n=15. Error bars indicate SEM.

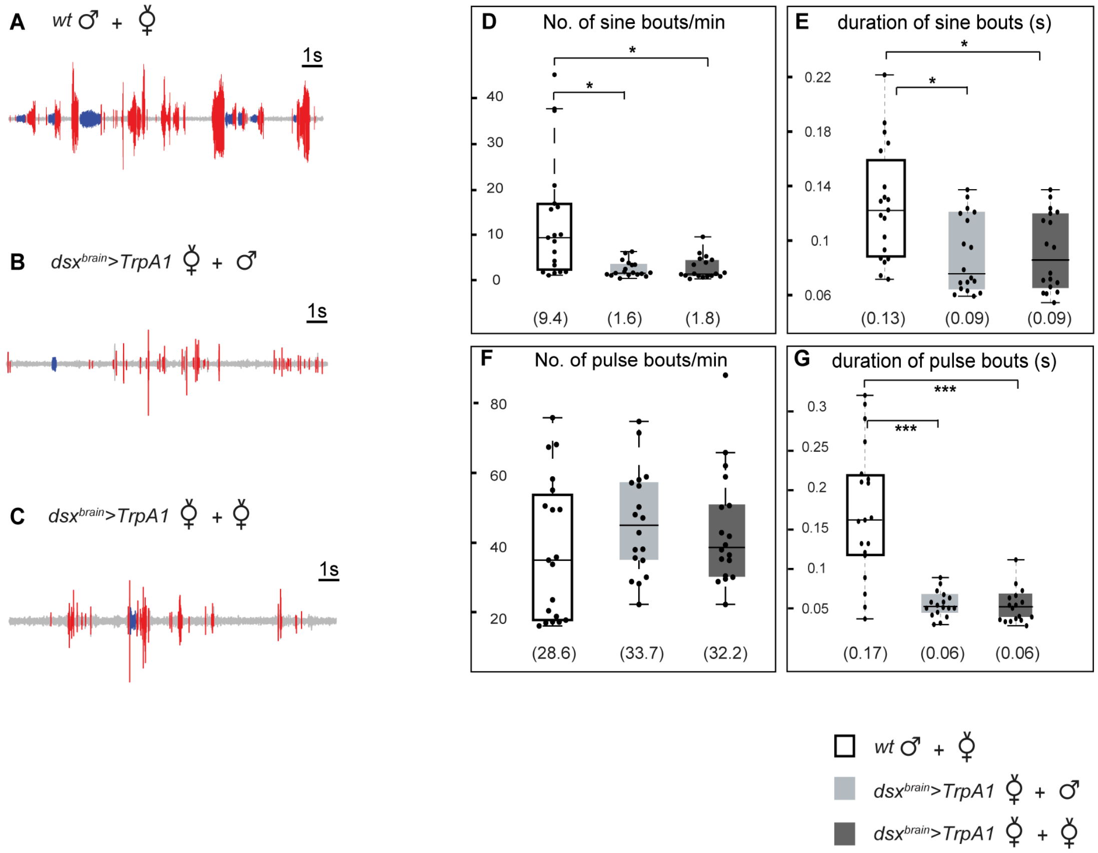

Figure S2, related to Figure 2. Characterization of song events and bout structure in *dsx^brain>TrpA1* females. (**A**-**C**) Courtship song was detected [S1] in recordings of 10 min. Sine song is shown in blue and pulses in red. (**A**) Overview of courtship song trace produced by a wild-type male courting a wild-type virgin female at 33°C. (**B**,**C**) Overview of courtship song traces produced by a *dsx^brain>TrpA1* female paired with a wild-type male (**B**) or female (**C**) at 33°C. Timescale indicated in seconds (s). (**D**-**G**) Box plots of No of sine bouts/minute (**D**), duration of sine bouts (s) (**E**), No of pulse bouts/minute (**F**) and duration of pulse bouts (s) (**G**). Median with interquartile range is indicated by boxes. Mean values are indicated below each box. n= 19-20 flies recorded. Kruskal-Wallis ANOVA was performed in all assays (*p < 0.01; ***p < 0.0001).

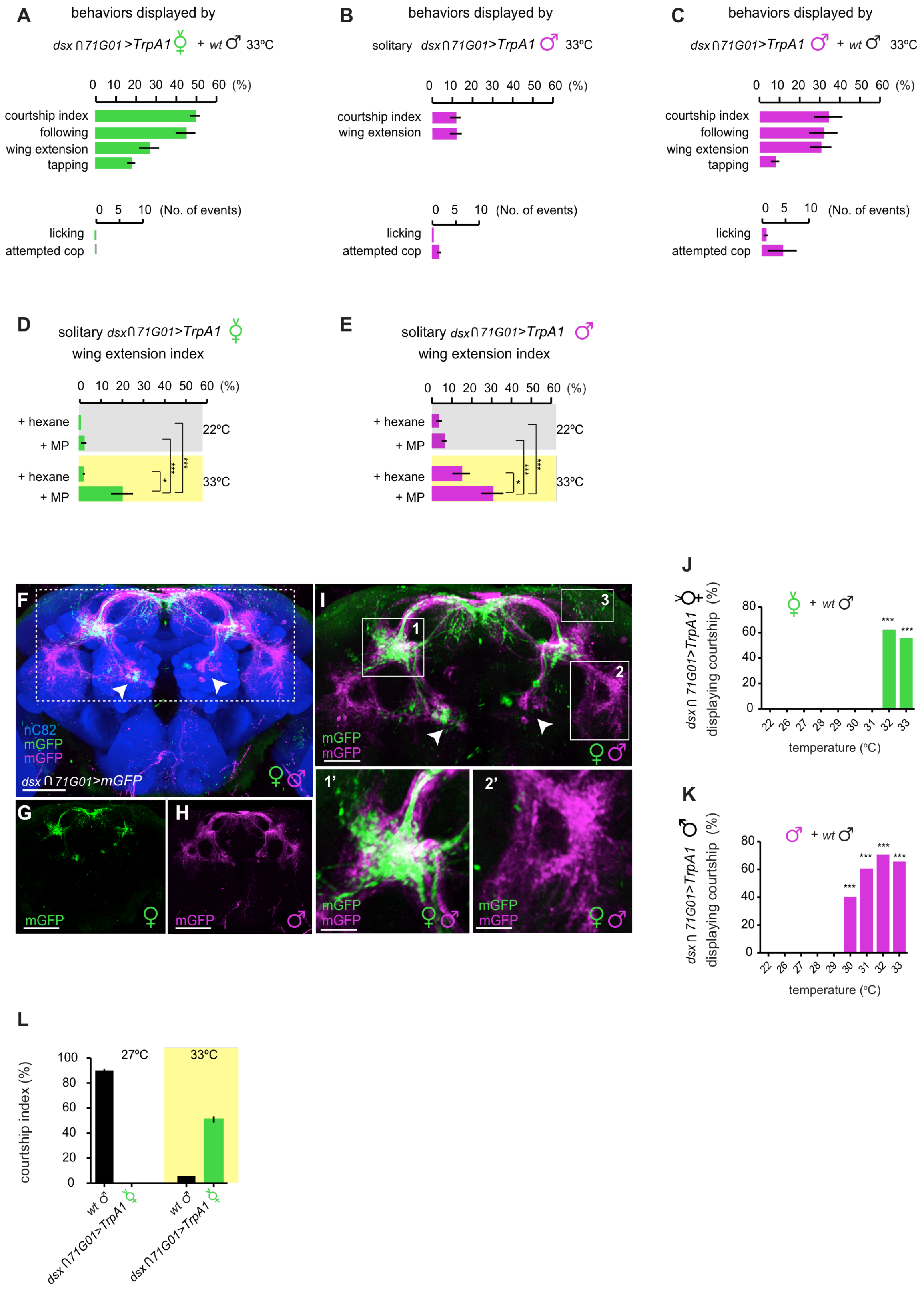
A
behaviors displayed by
dsx ∩ 71G01 >TrpA1 + wt 33ºC
0 10 20 30 40 50 60 (%)
courtship index
following
wing extension
tapping
0 5 10 (No. of events)
licking
attempted cop
B
behaviors displayed by
solitary dsx ∩ 71G01>TrpA1 33ºC
courtship index
wing extension
licking
attempted cop
C
behaviors displayed by
dsx ∩ 71G01>TrpA1 + wt 33ºC
courtship index
following
wing extension
tapping
licking
attempted cop
D
solitary dsx ∩ 71G01>TrpA1
wing extension index
+ hexane
+ MP
22ºC
33ºC
E
solitary dsx ∩ 71G01>TrpA1
wing extension index
F
nC82
mGFP
mGFP
dsx ∩ 71G01>mGFP
G
H
I
1
2
3
1'
2'
J
dsx ∩ 71G01>TrpA1 displaying courtship (%)
+ wt
temperature (ºC)
K
L
courtship index (%)
27ºC
33ºC
wt
dsx ∩ 71G01>TrpA1

Figure S3, related to Figure 3. Anatomical and behavioral characterization of *dsx*-pC1 neurons in females and males. (**A**-**C**) Behavioral assays were recorded at higher magnification to monitor individual courtship steps displayed either by *dsx∩71G01>TrpA1* virgin females paired with wild-type males (**A**), *dsx∩71G01 >TrpA1* males paired with wild-type males (**B**) or solitary *dsx∩71G01>TrpA1* males at 33°C (**C**). For following, wing extension and tapping the total time engaged in these steps was recorded; for licking and attempted copulation (attempted cop), it was the total number of events. n=12-15. Error bars indicate SEM. Genotype: *GMR71G01-lexA/UAS>stop>TrpA1;LexAop2-FLP/dsx*^{Gal4}. (**D**-**E**) Effects of methyl palmitate (MP) on courtship behavior. Wing extension index displayed by a solitary *dsx∩71G01>TrpA1* virgin female (**D**) or male (**E**) in the presence of hexane or MP at 22°C (grey box) or 33°C (yellow box). n=20. A Kruskal-Wallis ANOVA test was performed (*p < 0.05; ***p < 0.0001). Error bars indicate SEM. Wing extension index were measured over 3 minutes. (**F**-**I**) *dsx*-pC1 neurons are sexually dimorphic. (**F**) Confocal images of the female (**G**) and male (**H**) brains of *dsx∩71G01>mGFP* flies were registered onto an intersex template brain for *D. melanogaster* generated by the Jefferis lab [S2-S4] (https://zenodo.org/record/10591). mGFP staining in the female is shown in green (**F, G**) and mGFP staining in the male is shown in magenta (**F**,**H**). nc82 staining is shown in blue (**F**). Arrowheads indicate cell bodies. Scale bars: 25 µm (**F**) and 50 µm (**G**,**H**). Genotype: *GMR71G01-lexA/UAS>stop>myr::GFP;LexAop2-FLP/dsx*^{Gal4} (indicated as *dsx∩71G01>mGFP*). (**I**) Higher magnification of the dorsal brain shown in **F**. Arrowheads indicate cell bodies. Note that *dsx*-pC1 neurons show common arborizations in the brain of males and females (*e.g.,* region box 1), as well as male-specific innervations (*e.g.,* region box 2) and female-specific innervations (*e.g.,* region box 3). Scale bar: 25 µm. Higher magnification of region box 1 and 2 are shown in the lower panels (1' and 2'). Scale bars: 12,5 µm. (**J**-**K**) Characterization of the minimal temperature required in *dsx∩71G01>TrpA1* females (**J**) and males (**K**) to display courtship behaviors in the presence of a wild-type male. The graphs depict percentage of flies displaying courtship at increasing temperatures. Statistical comparisons of the indicated genotypes at different temperatures were made against genotypes at 22°C (Fisher exact Test; ***p < 0.0001). n=15-20. (**L**) Courtship assays for *dsx∩71G01>TrpA1* virgin females paired with wild-type males at 27°C or 33°C. The courtship indices displayed by both males and females are reported. The courtship levels displayed by *wt* males at 33°C are significantly different from those at 27°C, and the courtship levels displayed by *dsx∩71G01>TrpA1* females at 33°C are significantly different from those at 27°C (Kruskal-Wallis ANOVA; p< 0.0001). *UAS>stop>TrpA1/+; dsx*^{Gal4}/+ virgin females and *LexAop-FLP/+;71G01-LexA/+* virgin females did not display male-like courtship at 27°C or 33°C (data not shown). No significant differences in the courtship levels displayed by *wt* males paired with either *UAS>stop>TrpA1/+; dsx*^{Gal4}/+ control virgin females or *LexAop-FLP/+;71G01-LexA/+* control virgin females were found between 27°C and 33°C (data not shown). n=15-22. Error bars indicate SEM.

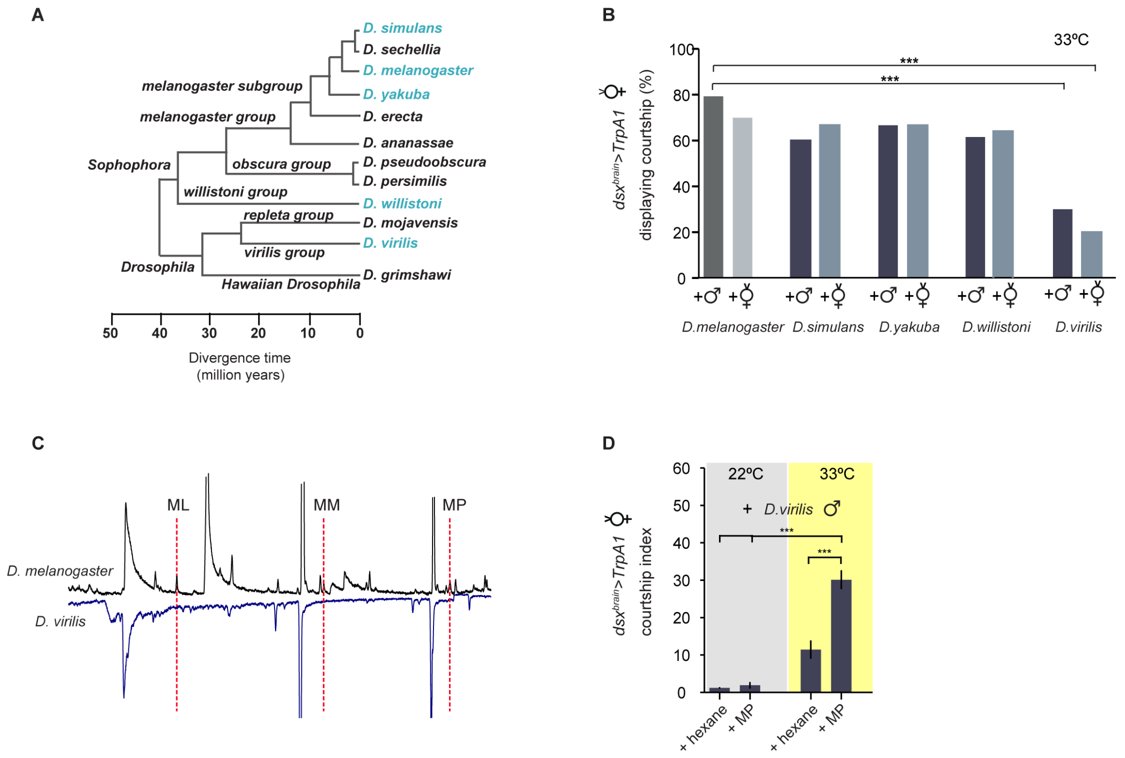

Figure S4, related to Figure 4. Activation of female brain *dsx*$^{+}$ neurons triggers male-courtship behaviors towards other *Drosophila* species.

(**A**) Schematic depicting phylogenetic relationships and estimated divergence times among species in the genus *Drosophila*. Modified from http://arthropods.eugenes.org/DroSpeGe/. (**B**) Percentage of *D. melanogaster Otd-FLP/UAS>stop>TrpA1;dsx*^{Gal4}*/+* virgin females (indicated as *dsx*^{brain}*>TrpA1*) displaying male-like courtship behaviors when paired with other *Drosophila* species at 33°C. A Fisher exact Test was performed (***$p < 0.0001$). n=15-20. (**C**) Representative TDU-GC-MS traces of individual *D. melanogaster* and *D. virilis* males. Methyl laurate (ML), methyl myristate (MM) and methyl palmitate (MP) are shown. None of these methyl pheromones are present in D. virilis males or females (data not shown). **(D)** Courtship index displayed by *dsx*^{brain}*>TrpA1* virgin females in the presence of *D.virilis* males perfumed with either hexane or methyl palmitate (MP) at 22°C or 33°C. Kruskal-Wallis ANOVA was performed (*** $p < 0.0001$). n=20-30. Error bars indicate SEM.

Intersection	Genotype	Number of *dsx*[+] neurons in females				
		pC1	pC2	pC3	aDN	Abg
dsx	*dsx^{Gal4}/UAS-mGFP*	10.6 ± 0.4	12.8 ± 0.5	6.6 ± 0.1	1.7 ± 0.3	311.3 ± 4.2
dsx∩Otd	*Otd-FLP/UAS>stop>myr::GFP; dsx^{Gal4}/+*	7.8 ± 0.2	11.6 ± 0.2	4.4 ± 0.3	0 ± 0	2.3 ± 2.4
dsx∩40F04(L)	*54785 (GMR40F04-lexA)/UAS>stop>myr::GFP; LexAop2-FLP/dsx^{Gal4}*	4.9 ± 1.1	10.6 ± 2.0	0 ± 0	0 ± 0	35.3 ± 1.2
dsx∩ 40F04(G)	*50094(GMR40F04-Gal4)/UAS>stop>myr::GFP; dsx^{FLP}/+*	5.1 ± 1.0	4.4 ± 1.3	0 ± 0	0 ± 0	2.6 ± 2.4
dsx∩71G01	*54733 (GMR71G01-lexA)/UAS>stop>myr::GFP; LexAop2-FLP /dsx^{Gal4}*	4.4 ± 0.8	0 ± 0	0 ± 0	0 ± 0	26.5 ± 2.5
dsx∩41A01	*54787 (GMR41A01-LexA)/UAS>stop>myr::GFP; LexAop2-FLP/dsx^{Gal4}*	1.2 ± 0.4	0 ± 0	5.7 ± 0.9	0 ± 0	13.3 ± 3
dsx∩42B01	*42B01-Gal4/ UAS>stop>myr::GFP; dsxFLP/+*	0 ± 0	8.4 ± 2.9	1.2 ± 0.4	0 ± 0	0 ± 0

Intersection	Genotype	Number of dsx^+ neurons in males							
		**pC1**	**pC2**	**pC3**	**aDN**	**SN**	**TN1**	**TN2**	**Abg**
dsx	*dsx^{Gal4}/UAS-mGFP*	52.5 ± 3.5	61.8 ± 4.6	12.8 ± 1.4	1.6 ± 0.8	1.7 ± 0.8	6.5 ± 0.5	22.2 ± 0.7	262.4 ± 16.9
dsx∩Otd	*Otd-FLP/UAS>stop>myr::GFP; dsx^{Gal4}/+*	46.2 ± 7.2	56.2 ± 7.6	11.4 ± 1.1	1.7 ± 0.8	0 ± 0	0 ± 0	0 ± 0	0 ± 0
dsx∩40F04(L)	*54785 (GMR40F04-lexA)/UAS>stop>myr::GFP; LexAop2-FLP/dsx^{Gal4}*	19.4 ± 3.1	32.3 ± 6.9	0.03 ± 0.17	0 ± 0	0 ± 0	6.4 ± 3.0	0.4 ± 0.7	34.1 ± 6.1
dsx∩ 40F04(G)	*50094 (GMR40F04-Gal4)/UAS>stop>myr::GFP; dsx^{FLP}/+*	2.9 ± 1.1	4.7 ± 1.6	0 ± 0	0 ± 0	0 ± 0	1.6 ± 0.8	0 ± 0	6.1 ± 1.4
dsx∩71G01	*54733 (GMR71G01-lexA)/UAS>stop>myr::GFP; LexAop2-FLP /dsx^{Gal4}*	17.2 ± 1.6	0.9 ± 1.3	0 ± 0	0 ± 0	0 ± 0	5.2 ± 0.8	0 ± 0	15.5 ± 4.8
dsx∩41A01	*54787 (GMR41A01-LexA)/UAS>stop>myr::GFP; LexAop2-FLP/dsx^{Gal4}*	6.0 ± 2.9	5.3 ± 4.6	2.7 ± 2.9	0 ± 0	0 ± 0	7.6 ± 1.9	0.2 ± 0.4	12.2 ± 5
dsx∩42B01	*42B01-Gal4/ UAS>stop>myr::GFP; dsxFLP/+*	0 ± 0	4.5 ± 1.8	1 ± 0	0 ± 0	0 ± 0	0 ± 0	0 ± 0	0 ± 0

Table S1, related to Figure 3. Quantification of intersected *dsx*$^+$ neurons in the central nervous system.

Quantification of *dsx*$^+$ intersected neurons targeted using *dsx* enhancer-LexA or Gal4 lines. Counts represent the number of intersected *dsx*$^+$ neurons for each of the indicated clusters in the female and

male brain (per hemisegment) and ventral nerve cord, including the abdominal ganglion (Abg). Mean ± standard deviation is shown. Counts of *dsx*–positive neurons were obtained by annotating and counting each individually stained cell with anti-GFP through a series of optical sections within a stack. n= 12-15.

Supplemental Experimental Procedures

Fly stocks

All flies were raised on standard medium in a 12 hr light/12 hr dark cycle at 22°C or 25°C on standard medium at 50% relative humidity. Wild-type flies were obtained from a *Canton-S* (CS) strain. *UAS-StingerII, UAS-mCD8::GFP, UAS-hid* [S5], *tubP-Gal80^{ts}* [S6], *LexAop2-FLP, 54785* (*GMR40F04-lexA*), *54733* (*GMR71G01-lexA*), *54787* (*GMR41A01-LexA*)*, 50094-Gal4* (*GMR40F04-Gal4*) and *42B01-Gal4* [S7, S8] were obtained from the Bloomington stock centre. Other fly stocks used include *dsx^{Gal4}* [S9], *dsx^{FLP}* [S10], *Otd-nls:FLPo* [S11] (provided by D.J. Anderson, Caltech), *PromE(800)-Gal4* [12] (provided by J. Levine, University of Toronto), *D. virilis, D.willistoni, D.simulans* and *D.yakuba* (provided by M.J. Ritchie, University of St Andrews), *UAS>stop>myr::GFP* [S13] (provided by G. Rubin, Janelia Farm), *fru^{Gal4}* [S14], *UAS>stop>mCD8::GFP* [S15] and *UAS>stop>trpA1^{myc}* [S16] (provided by B. Dickson; Janelia Farm).

Immunohistochemistry

Flies were reared at 25°C and aged for 4–6 days prior to dissection and staining. Samples were fixed in 4 % paraformaldehyde in Phosphate Buffered Saline (PBS) for 20 minutes at room temperature, then washed three times for 15 mins in PBT (1 x PBS, 0.4 % Triton X-100), and incubated for 40 min in PTN (PBT, 5% normal goat serum; Scottish Diagnostics and Molecular Probes). The specimens were incubated at 4°C for 4-5 days in PTN containing the primary antibodies. Next, they were washed in PBT for 2-4 hr and incubated in PTN containing the appropriate secondary antibodies for 2 days at 4°C. They were washed in PBT for 2-4 hr. Stained specimens were mounted in VectaShield (Vector Lab) on Polylysine treated microscope slides (BDH). Primary antibodies used were: rabbit anti-GFP (1:1000, Invitrogen Molecular Probes, Carlsbad, CA), chicken anti-GFP (1:1000, Abcam), mouse mAb nC82 (1:10, DSHB, Univ. of Iowa, IA). Secondary antibodies used were: anti-rabbit Alexa Fluor488, and anti-mouse Alexa Fluor546 conjugates (1:300 Invitrogen Molecular Probes, Carlsbad, CA) and anti-chicken Alexa Fluor488 (1:500 Invitrogen Molecular Probes, Carlsbad, CA). Confocal stacks were taken with Olympus FV1000 and Leica SP5 microscopes. Images were processed in Amira 5.2 (Mercury Systems).

Brain image registration

Confocal images of the female and male brain of *GMR71G01-lexA)/UAS>stop>myr::GFP; LexAop2-FLP/dsx^{Gal4}* (*dsx∩R71G01)* were registered onto an intersex template brain for *D. melanogaster*

generated by the Jefferis lab, using a Fiji graphical user interface (GUI), as previously described [S2-S4] (https://zenodo.org/record/10591). Four male and female brain samples were analysed.

Behavioral assays

Flies were kept in vials in groups of 2 for 6–8 days before behavioral assays at 22°C or 25°C. Single-pair courtship tests [S17] were performed in round chambers (10 mm diameter × 4 mm height or 19 mm diameter × 4 mm height -for experiments in bigger chambers-) and recorded at different temperatures. Courtship index was measured as the proportion of time during 10 min that the female or male spent exhibiting any male-typical courtship behaviors (*e.g.,* following, wing vibration, tapping, licking, attempted copulation) upon courtship initiation. For tapping, following and wing extension, the total time engaged in these steps during 10 min was recorded; for licking and attempted copulation, it was the total number of events. For the courtship preference assay: a single *dsx^{brain}>TrpA1* female was placed in a chamber (19 mm diameter × 4 mm height) with a wild-type male and a wild-type female at 33°C. The proportion of time during 10 min that the *dsx^{brain}>TrpA1* female spent exhibiting any male-typical courtship behaviors towards the male or female was measured. In Figure 4H, Figure S3D-E and Figure S4D, courtship index was measured within 3 min to avoid evaporation of the candidate odors. In order to compare different behavioral assays, we also quantified courtship index and wing vibration index over 3 min upon courtship initiation in in Figure 4 B,D,F. For the experiments involving severed sensory modalities, tarsi, antennae, and maxillary palps were surgically removed bilaterally under anesthesia 1–3 days prior to behavioral testing, as per [S18]. The labellum or aristae were surgically removed under anesthesia 2–3 hr prior to behavioral testing, as per [S18]. To eliminate the contribution of visual cues, behavioral tests were carried out under far-red light. For cuticular compounds assays in Figure 4H or S3D-E, candidate compounds were diluted in hexane, applied onto a whatman filter paper and left for 5 minutes at 33°C to allow the solvent to evaporate. Methyl palmitate (Sigma) and methyl myristate (Sigma) were used at 1:10 (as per [S19]), whereas methyl laurate (Sigma) was tested at 1:1000. Ten µl of concentrated cVA (Cayman chemicals) was tested. Solitary females were behaviorally assessed in courtship chambers containing the filter paper with either hexane or candidate compounds at 22°C or 33°C. For the oenocyte-less experiments, adults lacking oenocytes were obtained from the progeny of the cross of +*: PromE(800)-Gal4, tubP-Gal80ts;+ to +: UAS-StingerII, UAS-hid/CyO;+*, following the protocol published in [S12]. For perfuming experiments (Figure S4D), 10µl of hexane was pippeted into a glass tube. After the solvent was evaporated, 10 flies were transferred to the glass tube and subjected to gentle pulses for a total of 5 minutes. The treated flies were then transferred to fresh food vials to recover and then tested for behavior. For experiments involving MP, after the initial procedure with hexane, the flies were transferred to a glass tube with MP (1:10 in hexane) and vortexed them for 1 minute before the behavioral assays. For experiments in Figure S3L, *dsx∩71G01>TrpA1(LexAopFlp/UAS>stop>TrpA1;dsx^{Gal4}/71G01-LexA)*, *UAS>stop>TrpA1/+; dsx^{Gal4}/*+ and *LexAop-FLP/+; dsx∩71G01-LexA/*+ virgin females paired with *wt* males were tested at 27°C or 33°C. In addition, to test the transition of behaviors upon increasing the

temperature the same *dsx∩71G01>TrpA1* females and *wt* males tested at 27°C were then evaluated at 33°C (data not shown).

TD-GC-MS.

Individual flies were placed in standard microvials in thermal desorption tubes and transferred using a GERSTEL MPS 2 XL multipurpose sampler into a GERSTEL thermal desorption unit (www.gerstel.de). After desorption at 200 °C for 5 min with solvent venting, the analytes were trapped in the liner of a GERSTEL CIS 4 Cooled Injection System at−50 °C, using liquid nitrogen for cooling. The components were transferred to the GC column by heating the programmable temperature vaporizer injector at 12 °C/s up to 210 °C and then held for 5 min. The GC-MS device (Agilent GC 7890A fitted with an MS 5975C inert XL MSD unit; www.agilent.com) was equipped with an HP5-MS UI column (19091S-433UI; Agilent Technologies) and operated as follows. The temperature of the gas chromatograph oven was held at 40 °C for 3 min and then increased by 5 grd/min to 200 °C and then by 20 grd/min to 260 °C, with the final temperature held for 15 min. For MS, the transfer line was held at 260 °C, the source was held at 230 °C, and the quad was held at 150 °C. Mass spectra were taken in EI mode (at 70 eV) in the range from 33 m/z to 500 m/z. The structures of most of the cuticular compounds were confirmed by comparison with reference compounds measured at the same conditions.

Song assays

For recording song, experimental flies were paired with a wild-type male or female in cylindrical courtship chambers with a diameter of 10 mm and a height of 4 mm. Sound was recorded with a CMP-5247TF-K microphone in an Insectavox [S20], that was modified by adding two peltier elements to its sides, that allow raising ambient temperature in the box to 33°C. Temperature was controlled with a National Instruments DAQ. Recordings were analyzed using the MATLAB toolbox FlySongSegmenter [S1], and additional custom-made MATLAB software (available upon request). All dsx^{brain}*>TrpA1* recordings were examined to ensure only females were singing. For pulse analysis we used the default settings of FlySongSegmenter. For sine analysis we considered sine trains exceeding a threshold of 0.2 of normalised amplitude (to exclude background noise).

Statistics

Behavioral means were compared using Kruskal-Wallis ANOVA or Mann-Whitney test and Dunn's post hoc statistical test were indicated. For Fisher's exact test, two-tail *p* values were compared with controls. Statistical analyses were performed with GraphPad Prism software (version 6.0b, SPSS Inc.).

Supplemental References

S1. Arthur, B.J., Sunayama-Morita, T., Coen, P., Murthy, M., and Stern, D.L. (2013). Multi-channel acoustic recording and automated analysis of *Drosophila* courtship songs. BMC Biol. *11*, 1741-7007.

S2. Cachero, S., Ostrovsky, A.D., Yu, J.Y., Dickson, B.J., and Jefferis, G.S. (2010). Sexual dimorphism in the fly brain. Curr. Biol. *20*, 1589-1601.

S3. Jefferis, G.S., Potter, C.J., Chan, A.M., Marin, E.C., Rohlfing, T., Maurer, C.R., Jr., and Luo, L. (2007). Comprehensive maps of *Drosophila* higher olfactory centers: spatially segregated fruit and pheromone representation. Cell *128*, 1187-1203.

S4. Ostrovsky, A., Cachero, S., and Jefferis, G. (2013). Clonal analysis of olfaction in *Drosophila*: image registration. Cold Spring Harb Protoc. *2013*, 347-349.

S5. Zhou, L., Schnitzler, A., Agapite, J., Schwartz, L.M., Steller, H., and Nambu, J.R. (1997). Cooperative functions of the reaper and head involution defective genes in the programmed cell death of *Drosophila* central nervous system midline cells. Proc Natl Acad Sci U S A *94*, 5131-5136.

S6. McGuire, S.E., Le, P.T., Osborn, A.J., Matsumoto, K., and Davis, R.L. (2003). Spatiotemporal rescue of memory dysfunction in *Drosophila*. Science *302*, 1765-1768.

S7. Zhou, C., Pan, Y., Robinett, C.C., Meissner, G.W., and Baker, B.S. (2014). Central brain neurons expressing *doublesex* regulate female receptivity in *Drosophila*. Neuron *83*, 149-163.

S8. Pan, Y., Meissner, G.W., and Baker, B.S. (2012). Joint control of *Drosophila* male courtship behavior by motion cues and activation of male-specific P1 neurons. Proc Natl Acad Sci U S A *109*, 10065-10070.

S9. Rideout, E.J., Dornan, A.J., Neville, M.C., Eadie, S., and Goodwin, S.F. (2010). Control of sexual differentiation and behavior by the *doublesex* gene in *Drosophila melanogaster*. Nat Neurosci. *13*, 458-466.

S10. Rezaval, C., Nojima, T., Neville, M.C., Lin, A.C., and Goodwin, S.F. (2014). Sexually dimorphic octopaminergic neurons modulate female postmating behaviors in *Drosophila*. Curr. Biol. *24*, 725-730.

S11. Asahina, K., Watanabe, K., Duistermars, B.J., Hoopfer, E., Gonzalez, C.R., Eyjolfsdottir, E.A., Perona, P., and Anderson, D.J. (2014). Tachykinin-expressing neurons control male-specific aggressive arousal in *Drosophila*. Cell *156*, 221-235.

S12. Billeter, J.C., Atallah, J., Krupp, J.J., Millar, J.G., and Levine, J.D. (2009). Specialized cells tag sexual and species identity in *Drosophila melanogaster*. Nature *461*, 987-991.

S13. Pfeiffer, B.D., Ngo, T.T., Hibbard, K.L., Murphy, C., Jenett, A., Truman, J.W., and Rubin, G.M. (2010). Refinement of tools for targeted gene expression in *Drosophila*. Genetics *186*, 735-755.

S14. Stockinger, P., Kvitsiani, D., Rotkopf, S., Tirian, L., and Dickson, B.J. (2005). Neural

circuitry that governs Drosophila male courtship behavior. Cell *121*, 795-807.

S15. Yu, J.Y., Kanai, M.I., Demir, E., Jefferis, G.S., and Dickson, B.J. (2010). Cellular organization of the neural circuit that drives *Drosophila* courtship behavior. Curr. Biol. *20*, 1602-1614.

S16. von Philipsborn, A.C., Liu, T., Yu, J.Y., Masser, C., Bidaye, S.S., and Dickson, B.J. (2011). Neuronal control of Drosophila courtship song. Neuron *69*, 509-522.

S17. Villella, A., Gailey, D.A., Berwald, B., Ohshima, S., Barnes, P.T., and Hall, J.C. (1997). Extended reproductive roles of the fruitless gene in *Drosophila melanogaster* revealed by behavioral analysis of new fru mutants. Genetics *147*, 1107-1130.

S18. Fan, P., Manoli, D.S., Ahmed, O.M., Chen, Y., Agarwal, N., Kwong, S., Cai, A.G., Neitz, J., Renslo, A., Baker, B.S., et al. (2013). Genetic and neural mechanisms that inhibit *Drosophila* from mating with other species. Cell *154*, 89-102.

S19. Dweck, H.K., Ebrahim, S.A., Thoma, M., Mohamed, A.A., Keesey, I.W., Trona, F., Lavista-Llanos, S., Svatos, A., Sachse, S., Knaden, M., et al. (2015). Pheromones mediating copulation and attraction in *Drosophila*. Proc Natl Acad Sci U S A *112*, E2829-2835.

S20. Gorczyca, M.H., J.C. (1987). The Insectavox, an integrated device for recording and amplifying courtship songs of *Drosophila*. Dros. Inf. Serv *66*, 4.
